# Supplementary material for: Shared and distinct mechanisms of UBA1 inactivation across different diseases
Source: EMBO J. 2024 Feb 15;43(10):1919–46. doi: 10.1038/s44318-024-00046-z (PMC11099125; doi:10.1038/s44318-024-00046-z)
Supplement: Supplementary file 3 — Appendix [file 44318_2024_46_MOESM3_ESM.pdf]

Appendix

Shared and Distinct Mechanisms of UBA1 Inactivation Across Different Diseases

Jason C. Collins[1], Samuel J. Magaziner[2,3], Maya English[1], Bakar Hassan[4], Xiang Chen[4], Nicholas Balanda[2,3], Meghan Anderson [2,3], Athena Lam [2,3], Sebastian Fernandez-Pol[5], Bernice Kwong[6], Peter L. Greenberg[7], Benjamin Terrier[8], Mary E. Likhite[2,3], Olivier Kosmider[9], Yan Wang[10], Nadine L. Samara[11], Kylie J. Walters[4], David B. Beck[2,3,12,*], Achim Werner[1,13,*]

1. Stem Cell Biochemistry Unit, National Institute of Dental and Craniofacial Research, National institutes of Health, Bethesda, MD
2. Center for Human Genetics and Genomics, New York University School of Medicine, New York, NY
3. Division of Rheumatology, Department of Medicine, New York University School of Medicine, New York, NY
4. Protein Processing Section, Center for Structural Biology, National Cancer Institute, National Institutes of Health, Frederick, MD
5. Department of Pathology, Stanford University Medical School, Stanford, California, USA
6. Department of Dermatology, Stanford University Cancer Center, Stanford, CA, USA
7. Division of Hematology, Stanford University Cancer Center, Stanford, California, USA
8. Department of Internal Medicine, Hôpital Cochin, Assistance Publique-Hôpitaux de Paris, Paris
9. Laboratory of Hematology, Hôpital Cochin, Assistance Publique-Hôpitaux de Paris, Paris.
10. Mass Spectrometry Facility, National Institute of Dental and Craniofacial Research, National institutes of Health, Bethesda, MD
11. Structural Biochemistry Unit, National Institute of Dental and Craniofacial Research, National institutes of Health, Bethesda, MD
12. Department of Biochemistry and Molecular Pharmacology, New York University School of Medicine, New York, NY
13. Correspondence: achim.werner@nih.gov

* These authors contributed equally

Table of Contents:

Appendix Figure S1: Non-canonical VEXAS mutations, SMA-associated mutations, or LCNIS-associated mutations do not markedly change UBA1 subcellular localization…**p2**

Appendix Figure S2: SMA-associated and a subset of LCINS-associated mutations render UBA1 activity thermolabile…**p3**

Appendix Figure S3: LCINS-associated UBA1 p.H643Y and p.Q724P form aberrant thioesters and exhibit a bottleneck in ubiquitin transfer to E2 enzymes…**p5**

Appendix Figure S4: Mechanistic profiling of SMA and LCINS mutations…**p7**

Appendix Figure S5: Profiling of SMA and LCINS mutations in CHO cells…**p9**

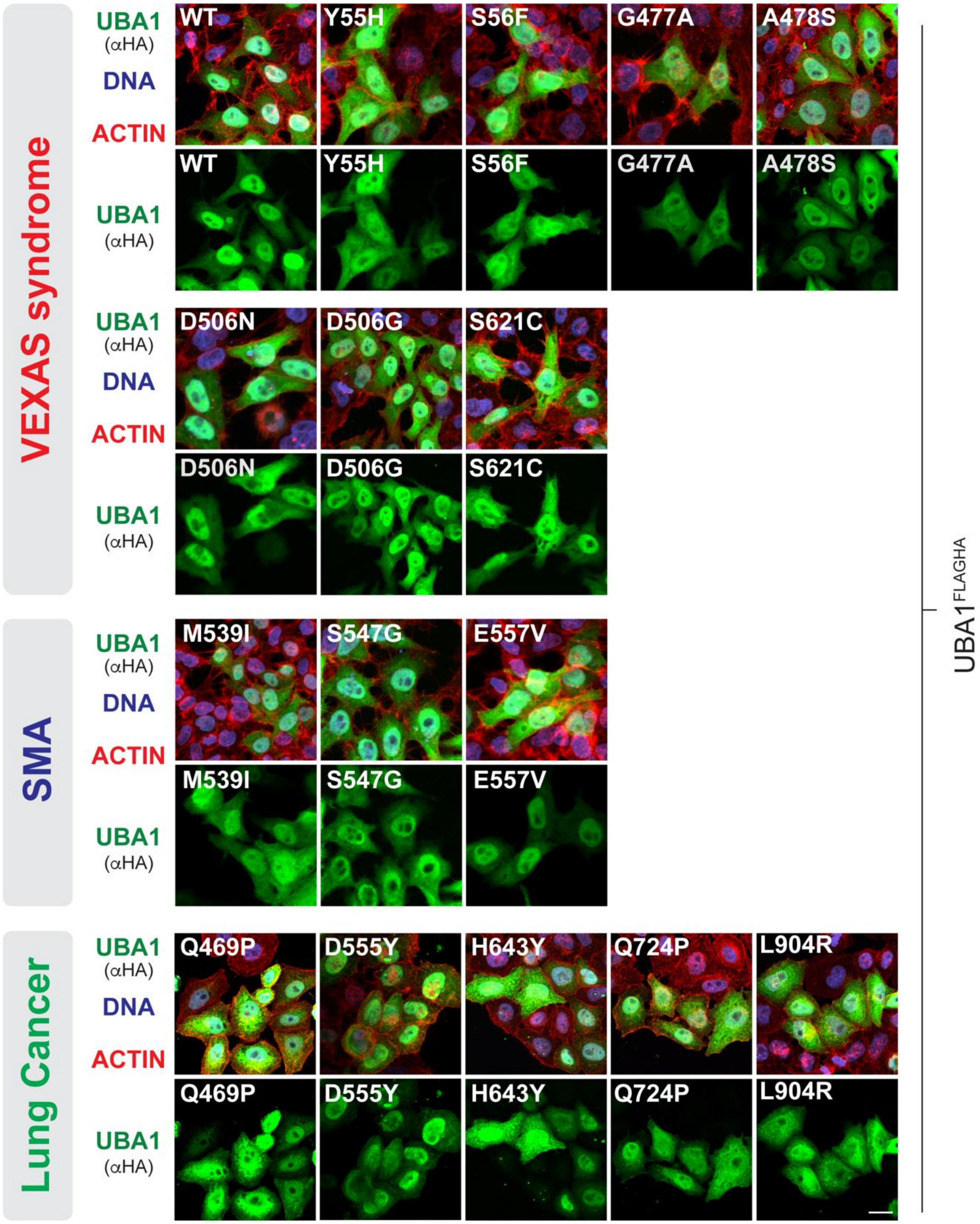

Appendix Figure S1: Non-canonical VEXAS mutations, SMA-associated mutations, or LCNIS-associated mutations do not markedly change UBA1 subcellular localization. HeLa cells were transfected with indicated UBA1$^{FLAG\text{-}HA}$ variants, fixed after 72h, and subjected to immunofluorescence microscopy using antibodies against HA and phalloidin and Hoechst to visualize ACTIN and DNA, respectively. Scale Bar = 20µm.

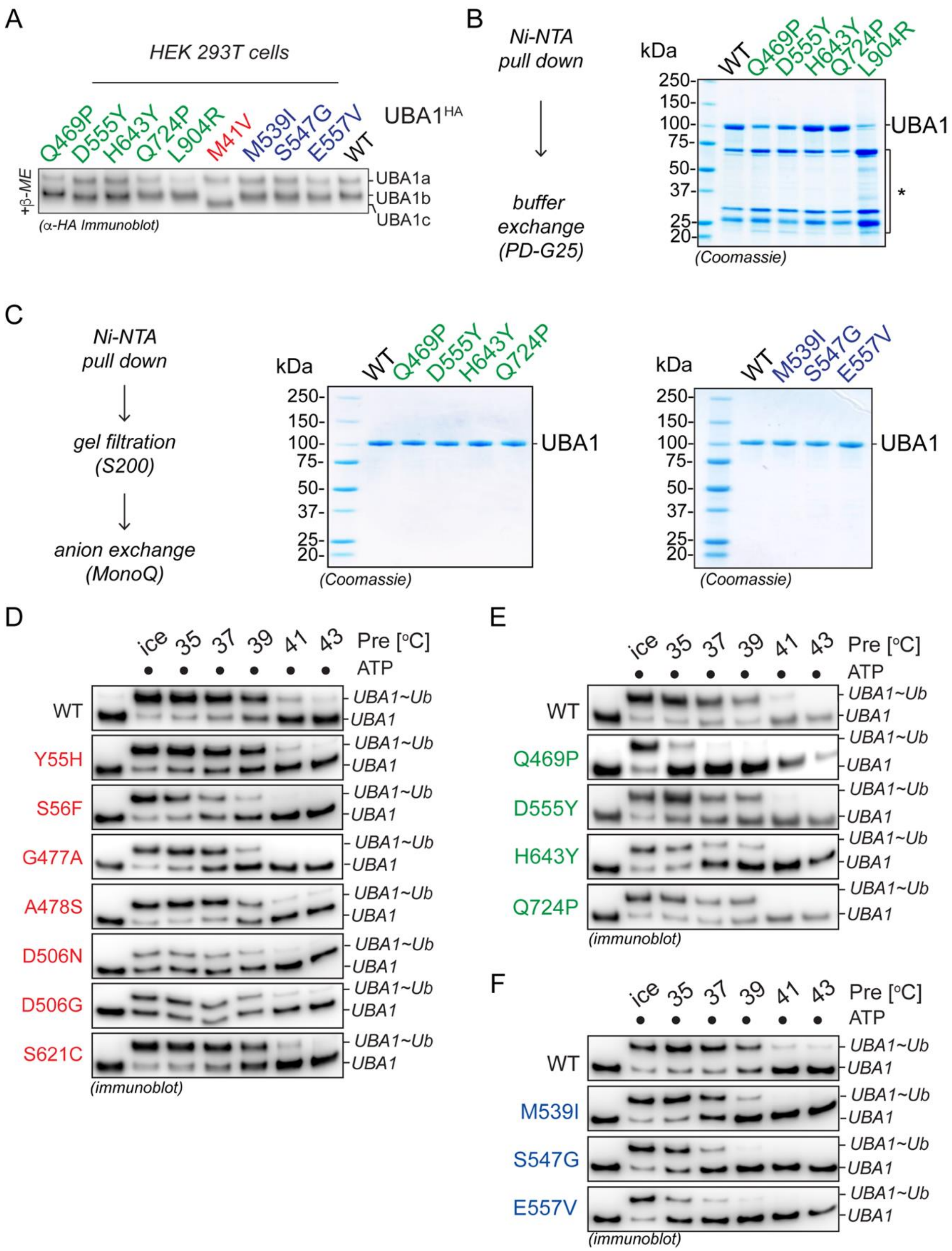

Appendix Figure S2: SMA-associated and a subset of LCINS-associated mutations render UBA1 activity thermolabile. A) SMA-associated mutations (blue) and LCINS-associated UBA1 mutations (green) do not lead to the cytoplasmic isoform swap from UBA1b to UBA1c, as evidenced by immunoblotting of HEK 293T cell lysates

transfected with the indicated UBA1 variants. **B**) Coomassie-stained SDS page gel of indicated LCINS-associated UBA1 variants (green) expressed and purified from *E.coli* via Ni-NTA pull down followed by buffer exchange via PD-G25 columns. This protocol only yielded ~30% pure UBA1 proteins but did allow for production of small amounts of the folding-defective UBA1 p.L904R variant for ubiquitin thioester formation and transthiolation studies. **C**) Coomassie-stained SDS page gel of indicated LCINS-associated UBA1 mutants (left, green) or SMA-associated UBA1 mutants (right, blue) expressed and purified from *E.coli* via Ni-NTA pull down, S200 gel filtration, and anion exchange chromatography, revealing highly protein purity. **D**) UBA1 p.S56F is the only non-canonical VEXAS mutation (red) that renders UBA1 thiolation thermolabile. Indicated UBA1 proteins were pre-incubated at varying denoted temperatures in the absence of substrate, subjected to ubiquitin thioester formation assays on ice using 500nM UBA1, 10μM ubiquitin, and 5mM ATP, and analyzed by anti-UBA1 immunoblotting. Quantifications are depicted in Figure 6D. **E**) LCINS mutations p.H643Y and p.Q724P (green) render UBA1 thiolation thermolabile. Indicated UBA1 proteins were treated as described in panel D. Quantifications are depicted in Figure 6E. **F**) All SMA-associated mutations (blue) render UBA1 thiolation thermolabile. Indicated UBA1 proteins were treated as described in panel D. Quantifications are depicted in Figure 6F.

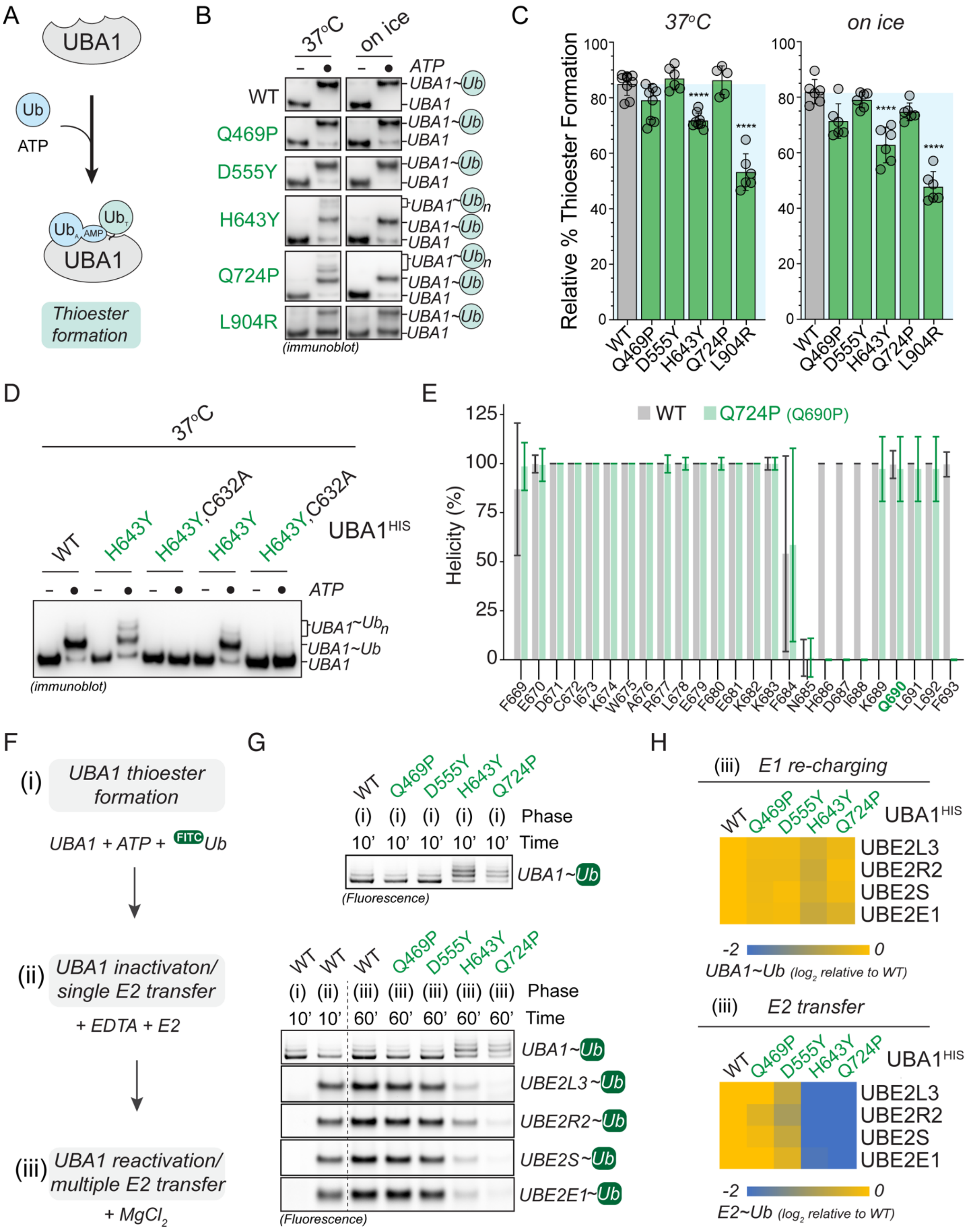

Appendix Figure S3: LCINS-associated UBA1 p.H643Y and p.Q724P form aberrant thioesters and exhibit a bottleneck in ubiquitin transfer to E2 enzymes. A) Schematic overview of the assay used to determine ubiquitin thioester formation activity

of UBA1. **B**) Non-reducing immunoblot analysis of *in vitro* ubiquitin thioester formation reactions carried out at 37°C (left panel) or on ice (right panel), revealing that of the tested LCINS-associated mutiations (green), UBA1 p.L904R is deficient in thiolation and that UBA1 p.H643Y and p.Q724P form aberrant thioesters at 37°C. **C**) Quantification of thioester formation (UBA1~Ub/total UBA1 signal) of the experiments shown in panel B. n = 5 – 8 biological replicates as indicated, error bars = s.d., **** = $p < 0.0001$, one-way ANOVA. **D**) Aberrant thioester formation of UBA1 p.H643Y and p.Q724P depends on the catalytic cysteine residue C632. Denoted recombinant UBA1b proteins (500 nM) were incubated with 10 μM ubiquitin and 5 mM ATP for 10 or 60 min at 37°C followed by anti-UBA1 immunoblot analysis. **E**) The average alpha helicity of residues in the helix containing Q724 (Q690) calculated over a 100 ns molecular dynamics trajectory for *S. cerevisiae* UBA1 (PDB: 4NNJ). Residues N-terminal to Q724 (Q690) in the wild-type protein (grey) maintain an average alpha helical content of 50% or greater. When the Q690P mutation (green) is modeled into the structure, the proline breaks the helix and residues (H686-I688) lose their alpha helicity. **F**) Schematic overview of the sequential, three-phase *in vitro* assay used to measure UBA1 transthiolation. (i) Complete charging of UBA1 by incubation of 250nM UBA1b with 10μM FITC-ubiquitin and 5mM ATP (ii) Quenching of UBA1 charging and single transfer to E2 enzyme by addition of 100mM EDTA and 1μM E2 enzyme (iii) Reactivation of UBA1 charging and multi transfer to E2 enzyme by addition of 100mM $MgCl_2$ **G**) LCINS mutations p.H643Y and p.Q724P but not p.Q469P or P.D555Y are deficient in E2 transthiolation *in vitro*. Indicated UBA1 proteins were subjected to the experiment described in panel F using either UBE2D3, UBE2R2, or UBE2S. Reactions were subjected to SDS page and analyzed by fluorescence imaging. *Upper panel*: Fluorescence scan showing UBA1 charging after reaction phase (i) as control. *Lower panel*: Fluorescence scan showing UBA1 re-charging and E2 transfer after reaction phase (iii). **H**) Quantifications of UBA1 ubiquitin thioester levels (*upper panel*) and E2 ubiquitin thioester levels (*lower panel*) after phase (iii) shown in panel G. E2 ubiquitin thioester levels in phase (iii) were first normalized to the total FITC signal in phase (i) and then normalized to WT. n = 3 biological replicates per condition.

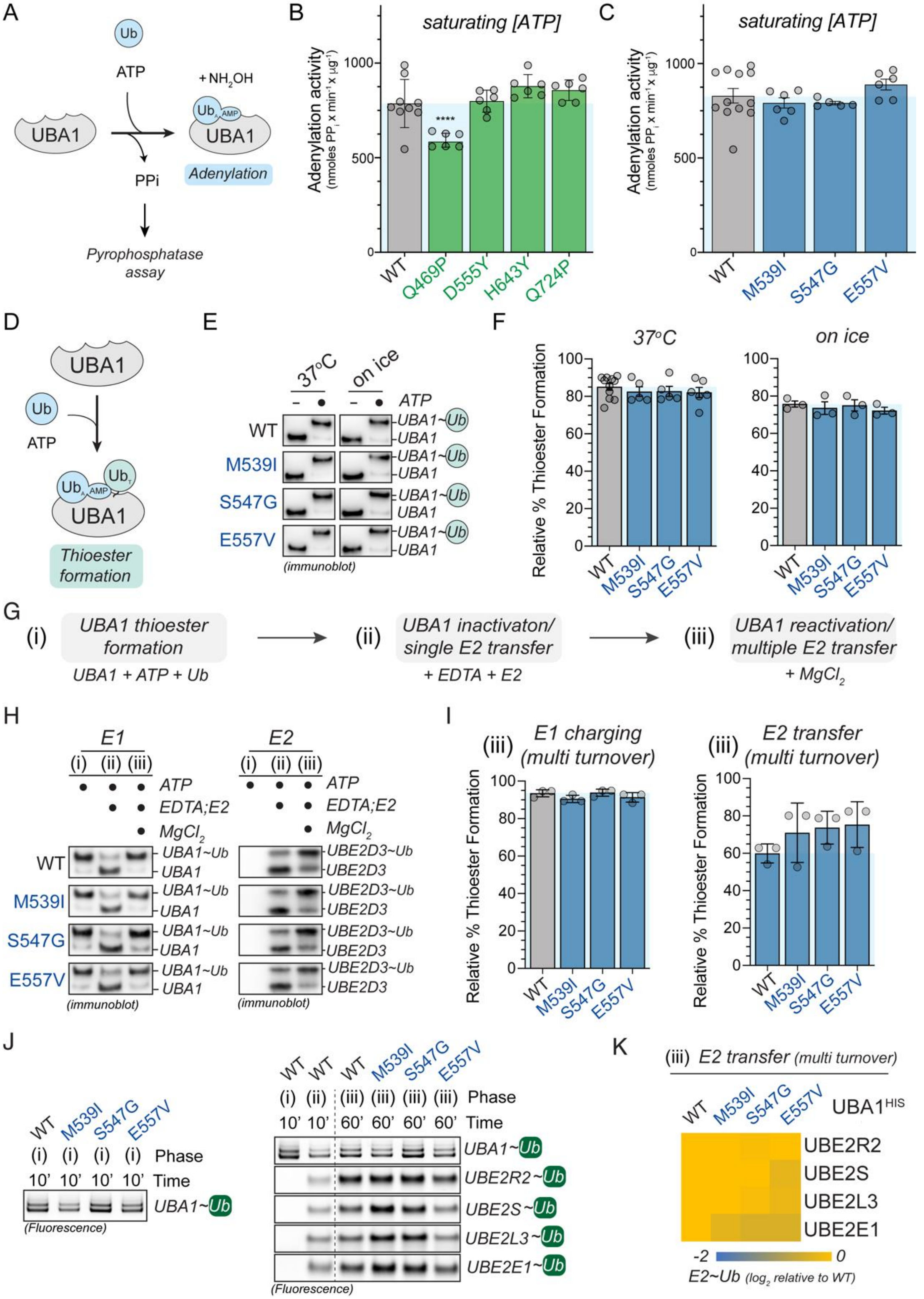

Appendix Figure S4: Mechanistic profiling of SMA and LCINS mutations. A) Schematic overview of the *in vitro* assay used to determine the efficiency of the first

adenylation reaction of UBA1. The assay measures the production of pyrophosphate (PP_i) and the reaction is carried out in presence of NH_2OH to reduce the ubiquitin thioester. **B**) Amongst LCINS mutations (green) only UBA1 p.Q469P confers a significant but small impairment of adenylation activity *in vitro* at saturating ATP and ubiquitin conditions. n = 2-3 biological replicates with 3 technical replicates each, error bars = s.d., **** = $p < 0.0001$, one-way ANOVA. **C**) SMA-associated UBA1 mutations (blue) have no obvious effect on adenylation activity *in vitro* at saturating ATP and ubiquitin conditions. n = 2-4 biological replicates with 3 technical replicates each, error bars = s.d., no significant changes observed, one-way ANOVA. **D**) Schematic overview of the assay used to determine ubiquitin thioester formation activity of UBA1. **E**) Non-reducing immunoblot analysis of *in vitro* ubiquitin thioester formation reactions carried out at 37^oC (left panel) or on ice (right panel) and containing 500nM indicated UBA1b proteins, 10μM ubiquitin, and 5mM ATP, revealing that SMA-associated mutations (blue) have no obvious impact on UBA1 thiolation *in vitro*. **F**) Quantification of thioester formation (UBA1~Ub/total UBA1 signal) of the experiments shown in panel E. n = 3 – 10 biological replicates as indicated, error bars = s.d., **** = $p < 0.0001$, one-way ANOVA. **G**) Schematic overview of the sequential, three-phase *in vitro* assay used to measure UBA1 transthiolation. (i) Complete charging of UBA1 by incubation of 250nM UBA1b with 10μM ubiquitin and 5mM ATP (ii) Quenching of UBA1 charging and single transfer to E2 enzyme by addition of 100mM EDTA and 1 μM UBE2D3 (iii) Reactivation of UBA1 charging and multi transfer to E2 enzyme by addition of 100mM $MgCl_2$. **H**) SMA-associated mutations (blue) have no obvious impact on E2 transthiolation *in vitro*. Immunoblot analysis of the sequential, three-phase *in vitro* assay described in panel G using antibodies against UBA1 (left) or UBE2D3 (right). **I**) Quantifications of relative UBA1 re-charging (UBA1~Ub/total signal, left graph) and E2 thioester levels (UBE2D3~Ub/total signal, right graph) of multi turnover reactions (phase iii) depicted in panel H. n = 3 biological replicates as indicated, error bars = s.d., no significant changes detected, one-way ANOVA. **J**) SMA-associated UBA1 mutations (blue) are not defective in E2 transthiolation *in vitro*. Indicated UBA1 proteins were subjected to the experiment described in panel G using either UBE2D3, UBE2R2, or UBE2S. Reactions were run on SDS page and analyzed by fluorescence imaging. *Left panel*: Fluorescence scan showing UBA1 charging after reaction phase (i) as control. *Right panel*: Fluorescence scan showing UBA1 re-charging and E2 transfer after reaction phase (iii). **K**) Quantifications of E2 ubiquitin thioester levels after phase (iii) shown in panel G. E2 ubiquitin thioester levels in phase (iii) were first normalized to the total FITC signal in phase (i) and then normalized to WT. n = 3 biological replicates per condition.

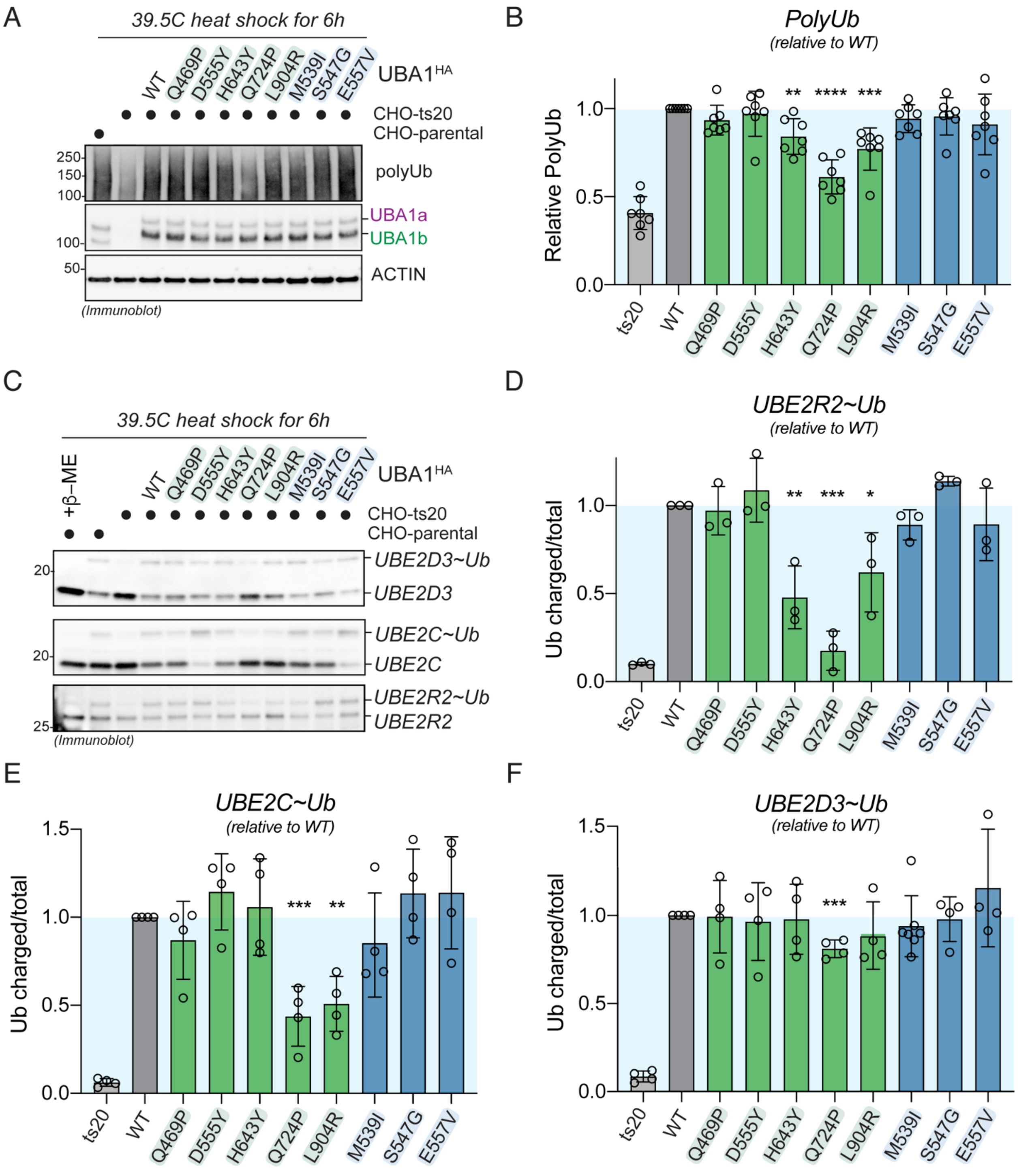

Appendix Figure S5: Profiling of SMA and LCINS mutations in CHO cells. A) Immunoblots of CHO ts20 cells reconstituted with indicated UBA1 variants and incubated at the permissive temperature for 6h, revealing that a subset of LCINS mutations (green), but not SMA-associated mutations (blue), are impaired in their ability to support ubiquitylation in cells. **B**) Quantification of global polyubiquitylation levels shown in panel A. n=7 biological replicates, error bars = s.d., ** = $p < 0.01$, *** = p <

0.001, **** = p < 0.0001, one-way ANOVA. **C)** Immunoblots of CHO ts20 cells reconstituted with indicated UBA1 variants and incubated at the permissive temperature for 6h, revealing that a subset of LCINS mutations are impaired in their ability to support E2 ubiquitin thioester levels in cells. **D-F**) Quantification of E2 charging levels (charged/total) shown in panel C. D) UBE2R2, E) UBE2C, F) UBE2D3. n=3 biological replicates, error bars = s.d., * = p < 0.05, ** = p < 0.01, *** = p < 0.001, **** = p < 0.0001, one-way ANOVA.
