# Supplementary figures and images for: Shared and distinct mechanisms of UBA1 inactivation across different diseases
Source: EMBO J. 2024 Feb 15;43(10):1919–46. doi: 10.1038/s44318-024-00046-z (PMC11099125; doi:10.1038/s44318-024-00046-z)

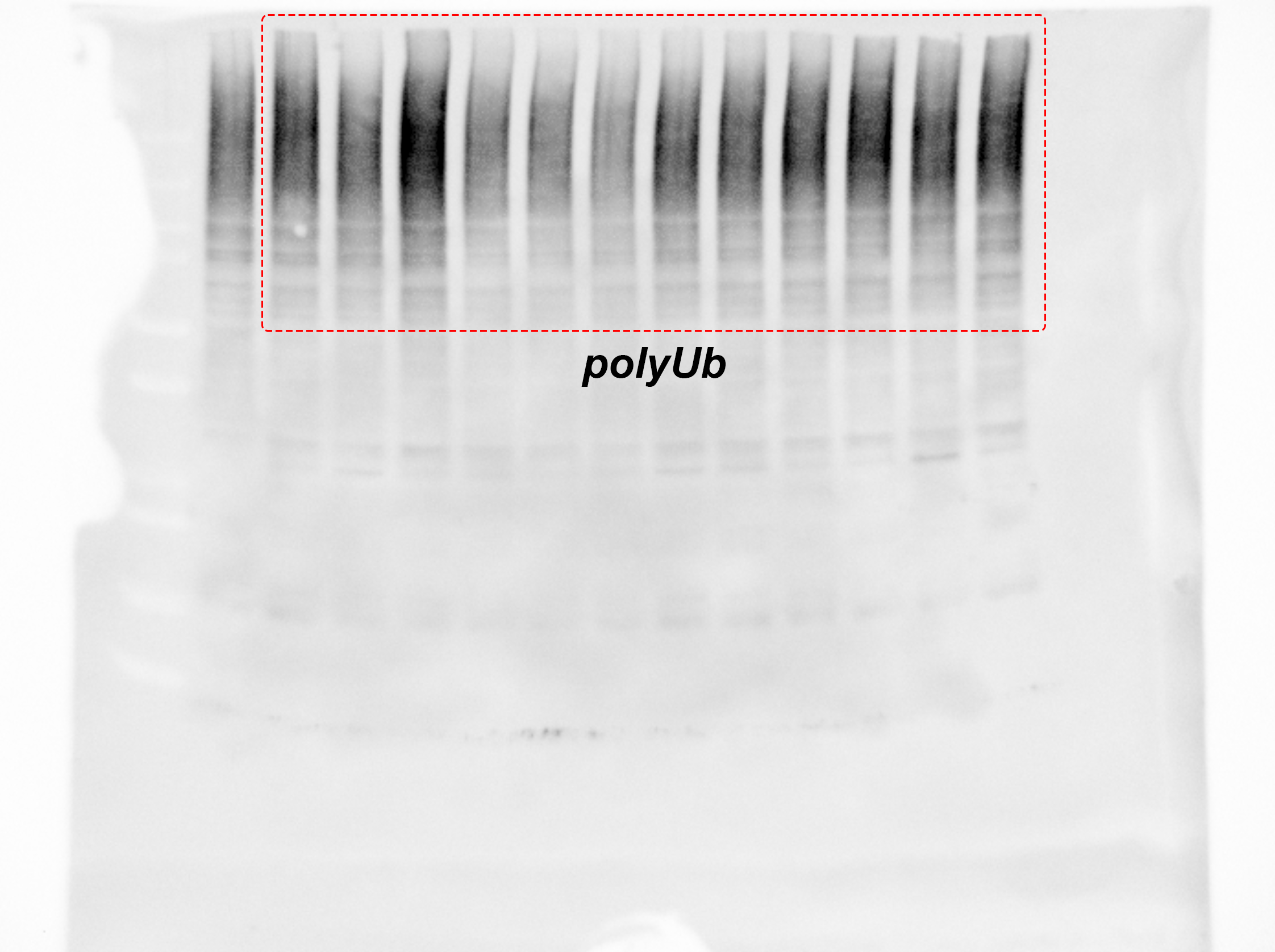

Supplement: Supplementary file 4 — Source Data Fig. 1 [file 44318_2024_46_MOESM4_ESM.zip › EMBOJ-2023-115688_Figure 1/1E/115688_SourceDATA_Fig1E_PolyUb.tif]

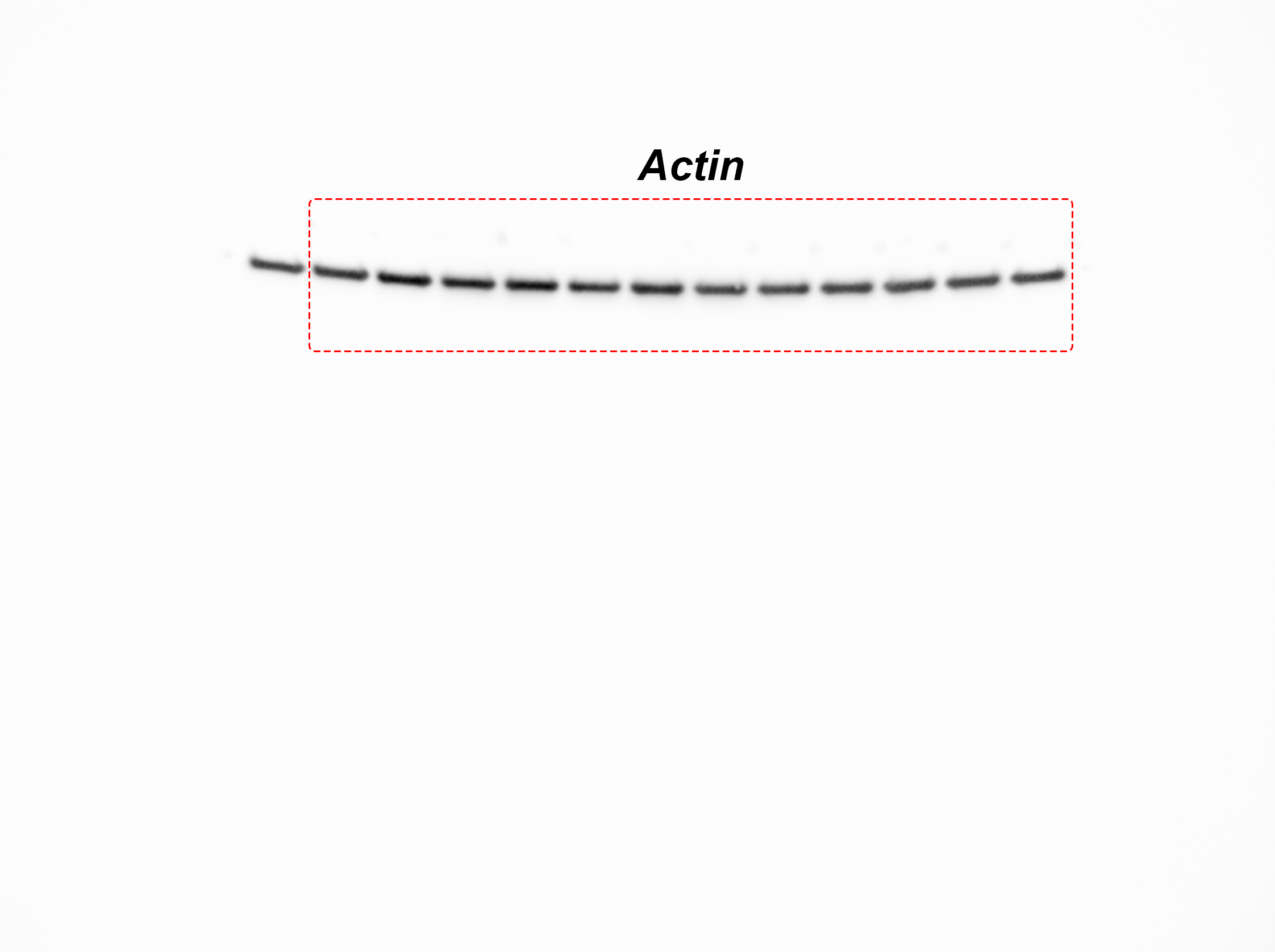

Supplement: Supplementary file 4 — Source Data Fig. 1 [file 44318_2024_46_MOESM4_ESM.zip › EMBOJ-2023-115688_Figure 1/1E/115688_SourceDATA_Fig1E_Actin.tif]

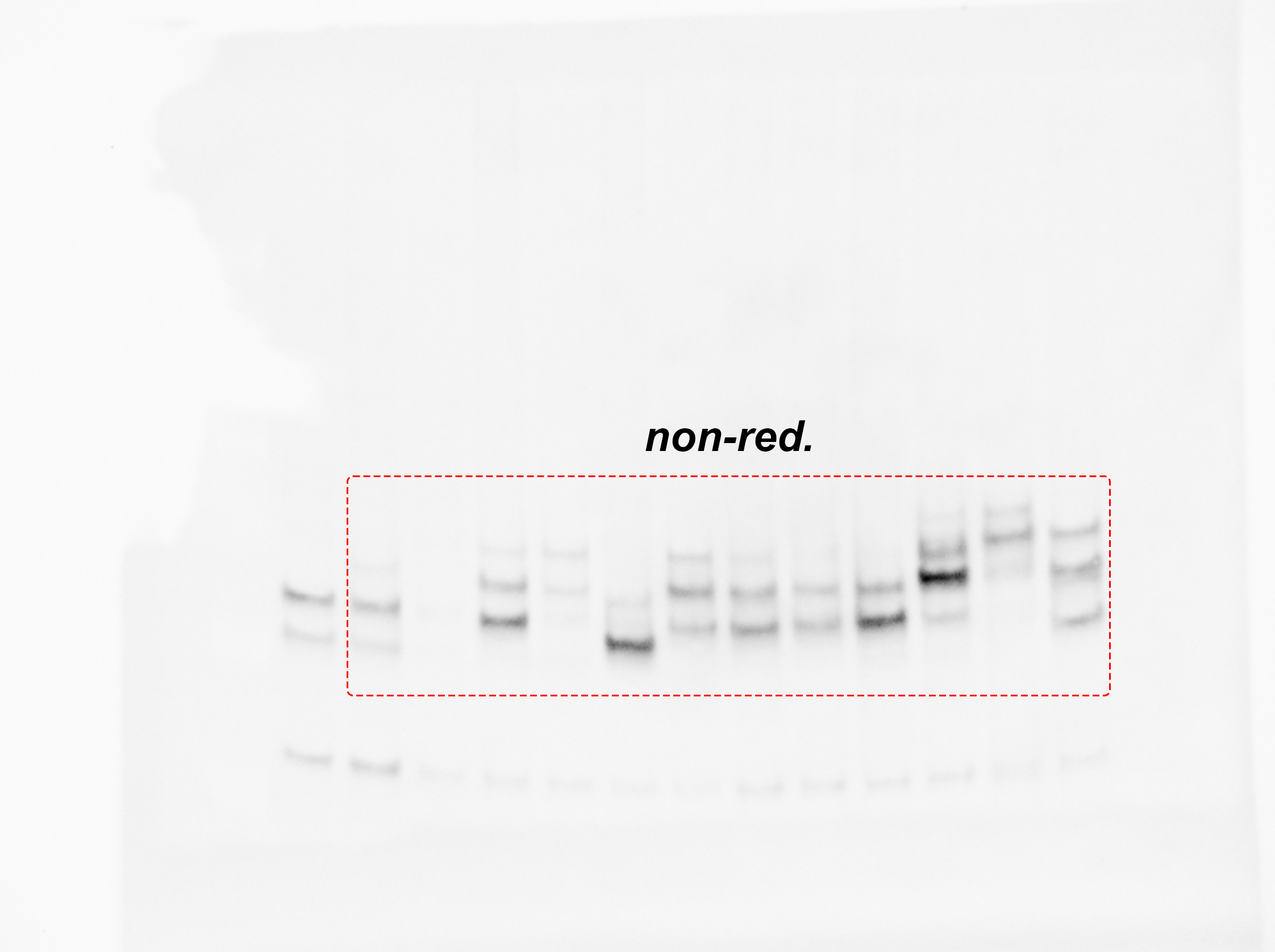

Supplement: Supplementary file 4 — Source Data Fig. 1 [file 44318_2024_46_MOESM4_ESM.zip › EMBOJ-2023-115688_Figure 1/1E/115688_SourceDATA_Fig1E_UBA1_Non-red.tif]

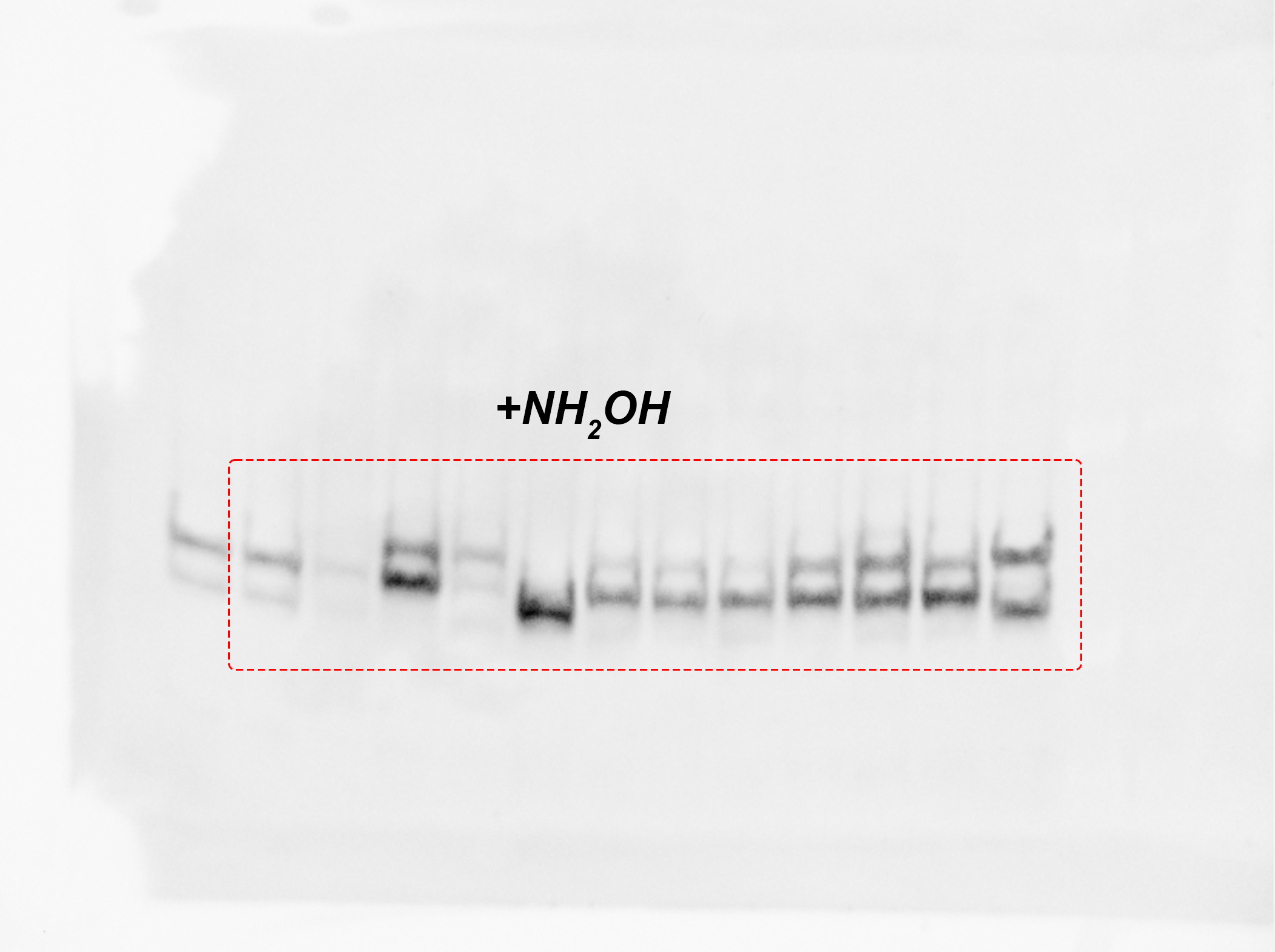

Supplement: Supplementary file 4 — Source Data Fig. 1 [file 44318_2024_46_MOESM4_ESM.zip › EMBOJ-2023-115688_Figure 1/1E/115688_SourceDATA_Fig1E_UBA1_NH2OH.tif]

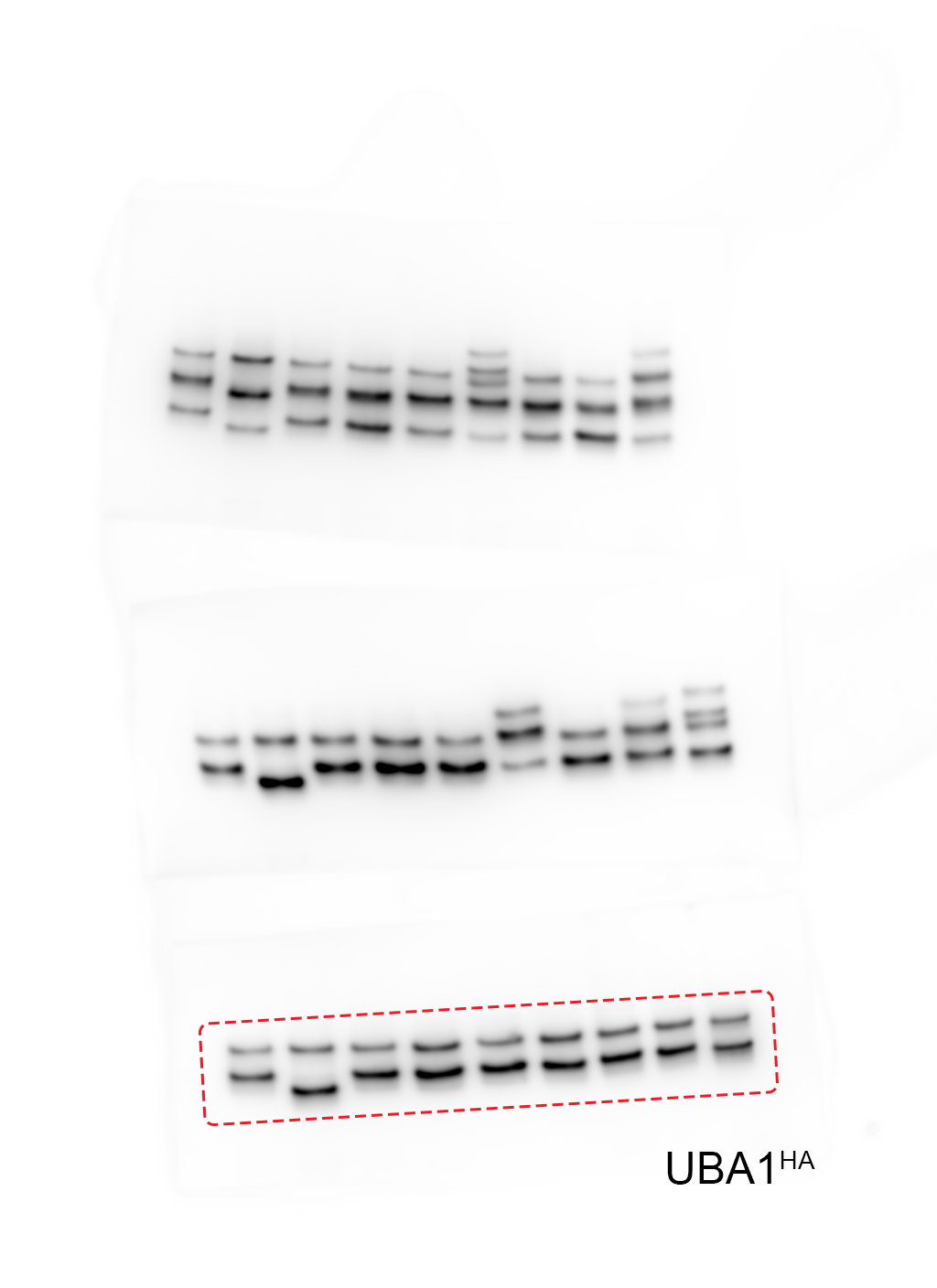

Supplement: Supplementary file 4 — Source Data Fig. 1 [file 44318_2024_46_MOESM4_ESM.zip › EMBOJ-2023-115688_Figure 1/1C/115688_SourceDATA_Fig1C_Hydroxylamine.tif]

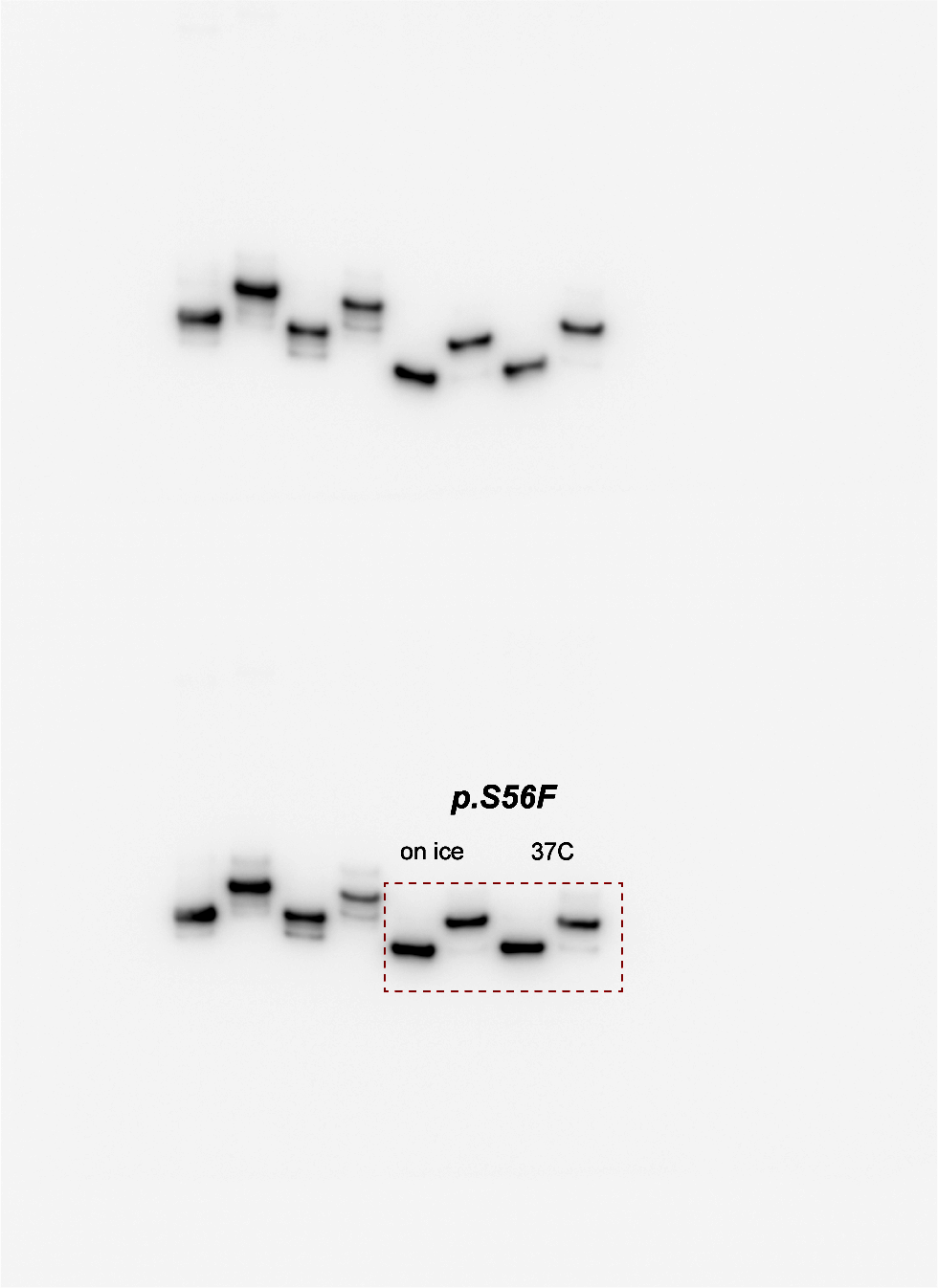

Supplement: Supplementary file 5 — Source Data Fig. 2 [file 44318_2024_46_MOESM5_ESM.zip › EMBOJ-2023-115688_Figure 2/2G/115688_SourceDATA_Fig2G_S56F.tif]

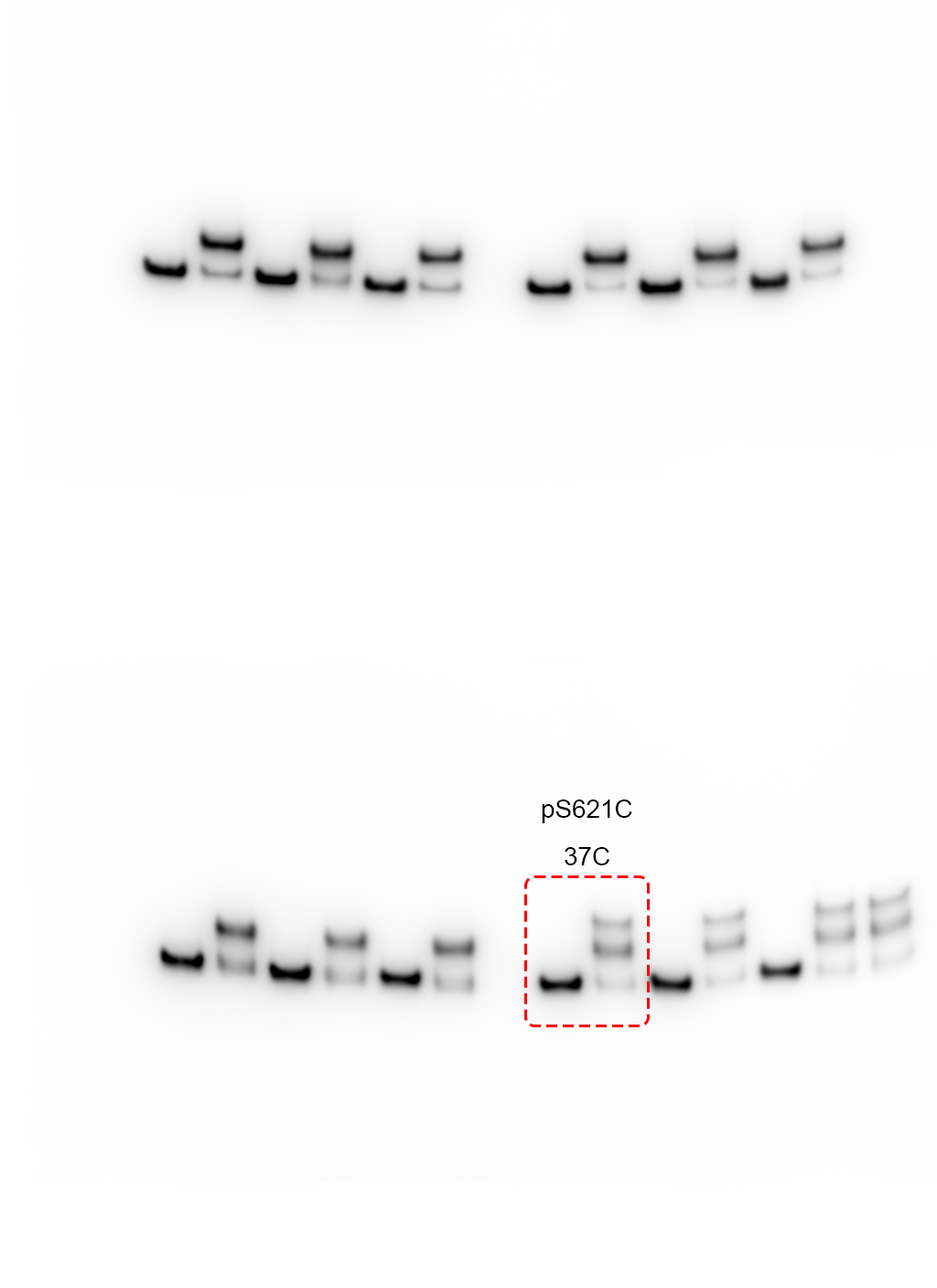

Supplement: Supplementary file 5 — Source Data Fig. 2 [file 44318_2024_46_MOESM5_ESM.zip › EMBOJ-2023-115688_Figure 2/2G/115688_SourceDATA_Fig2G_S621C_37C.tif]

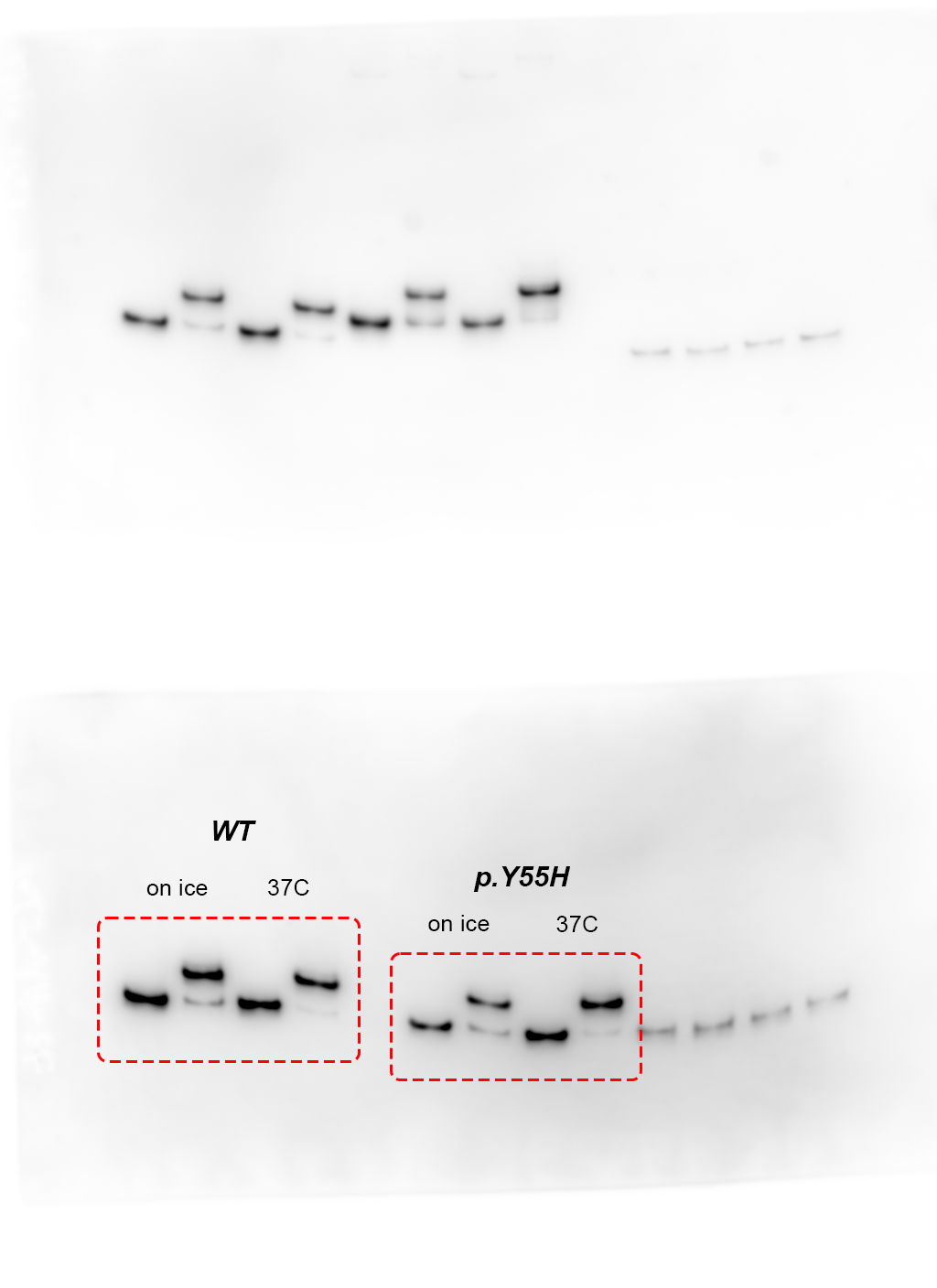

Supplement: Supplementary file 5 — Source Data Fig. 2 [file 44318_2024_46_MOESM5_ESM.zip › EMBOJ-2023-115688_Figure 2/2G/115688_SourceDATA_Fig2G_WT-Y55H.tif]

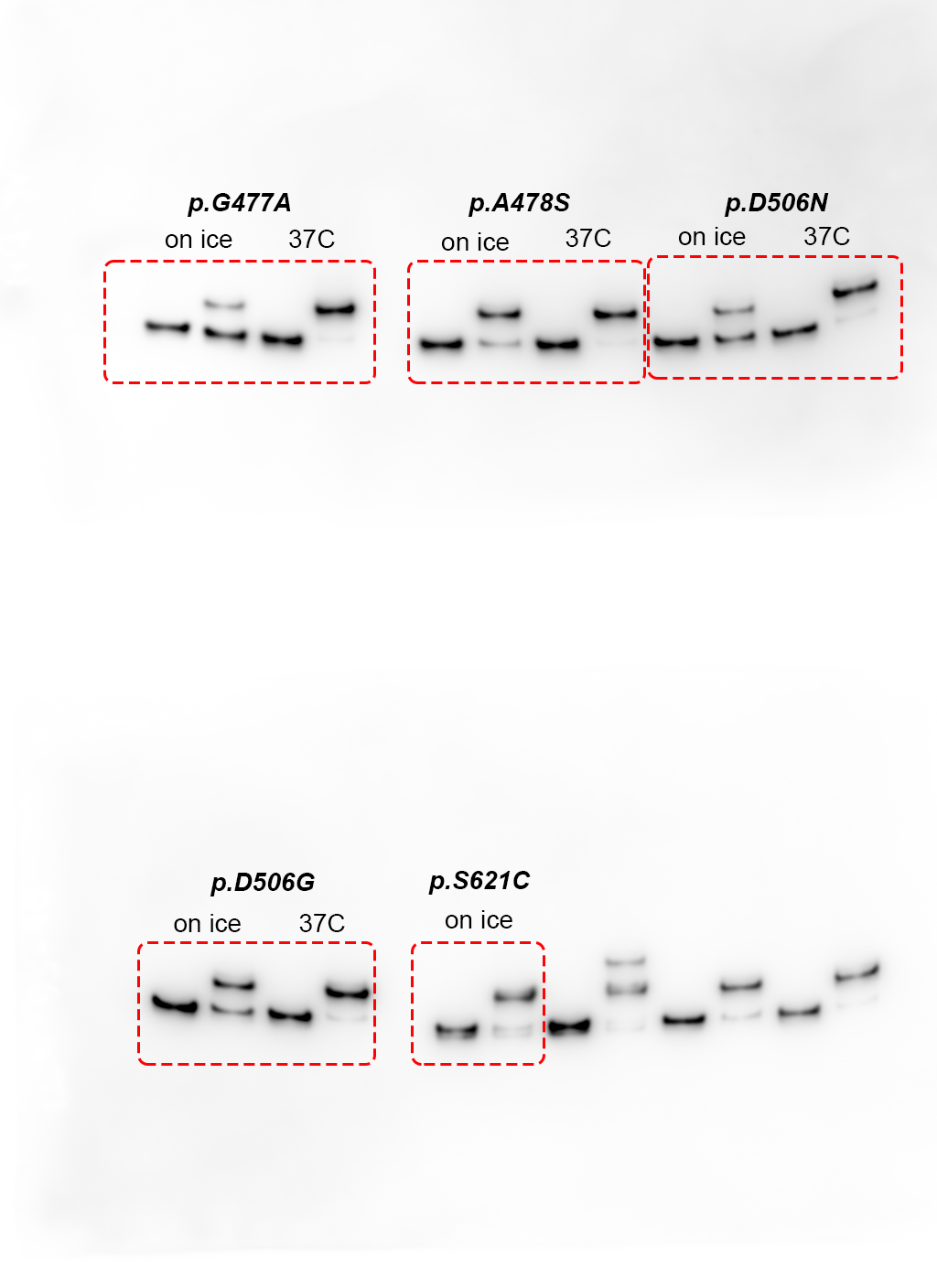

Supplement: Supplementary file 5 — Source Data Fig. 2 [file 44318_2024_46_MOESM5_ESM.zip › EMBOJ-2023-115688_Figure 2/2G/115688_SourceDATA_Fig2G_G477A-A478S-D506N-D506G-S621Cice.tif]

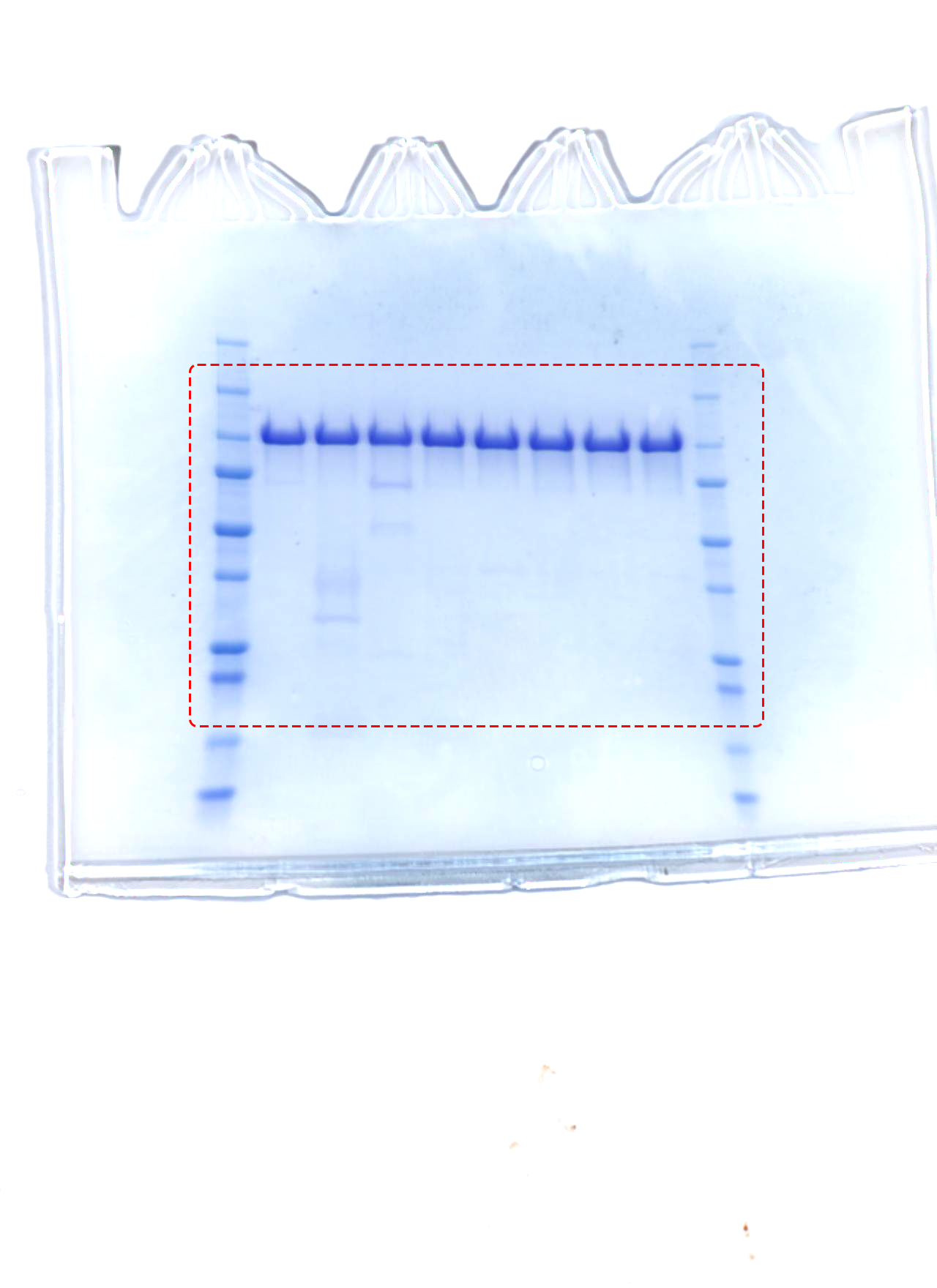

Supplement: Supplementary file 5 — Source Data Fig. 2 [file 44318_2024_46_MOESM5_ESM.zip › EMBOJ-2023-115688_Figure 2/2B/Coomassie_VEXAS_UBA1proteins_Fig2B.tif]

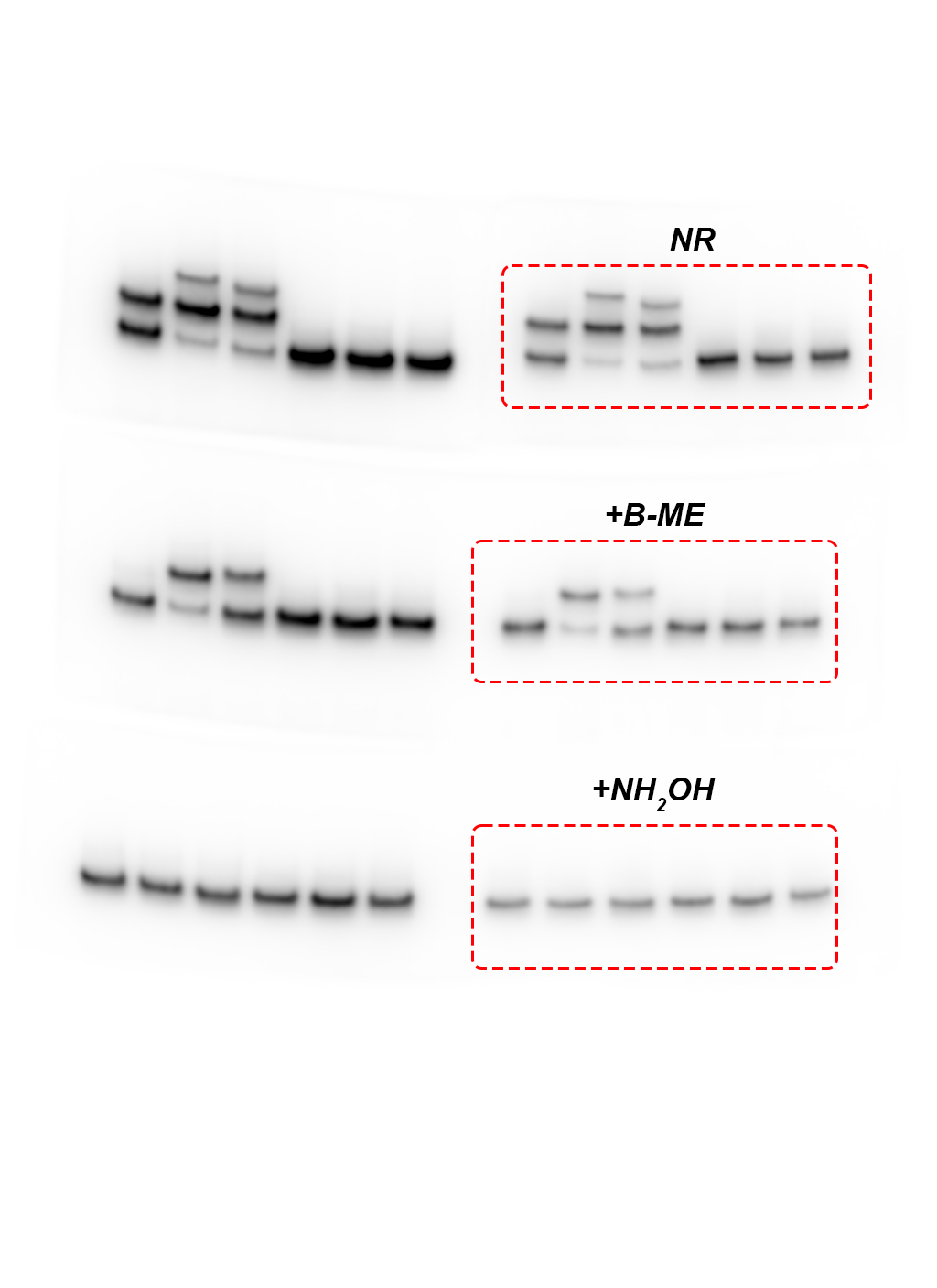

Supplement: Supplementary file 6 — Source Data Fig. 3 [file 44318_2024_46_MOESM6_ESM.zip › EMBOJ-2023-115688_Figure 3/3B/EMBOJ-2023-115688_SourceDATA_Fig3B_NR-BME-Hydroxylamine.tif]

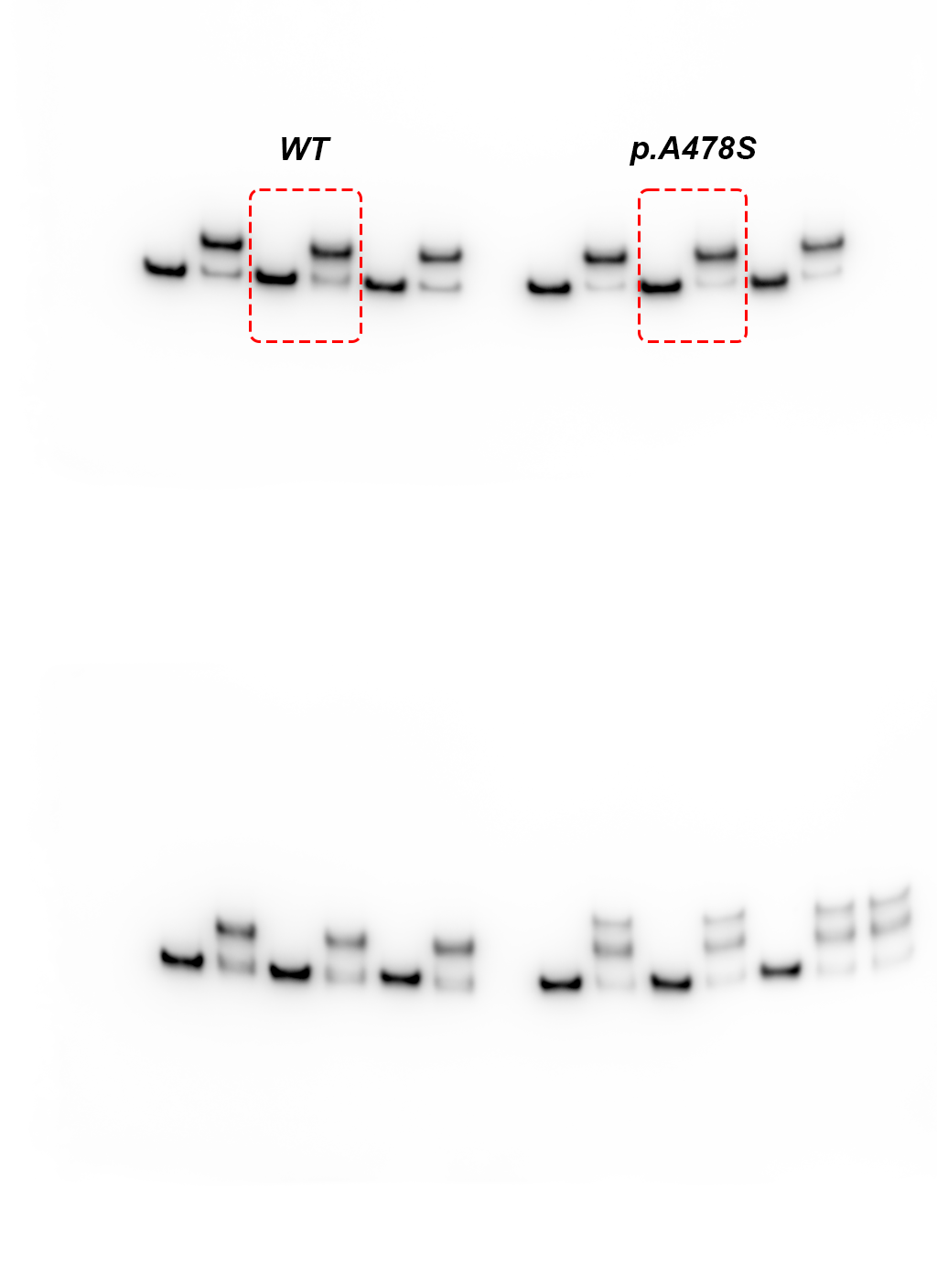

Supplement: Supplementary file 6 — Source Data Fig. 3 [file 44318_2024_46_MOESM6_ESM.zip › EMBOJ-2023-115688_Figure 3/3C/EMBOJ-2023-115688_SourceDATA_Fig3C_WT-A478S.tif]

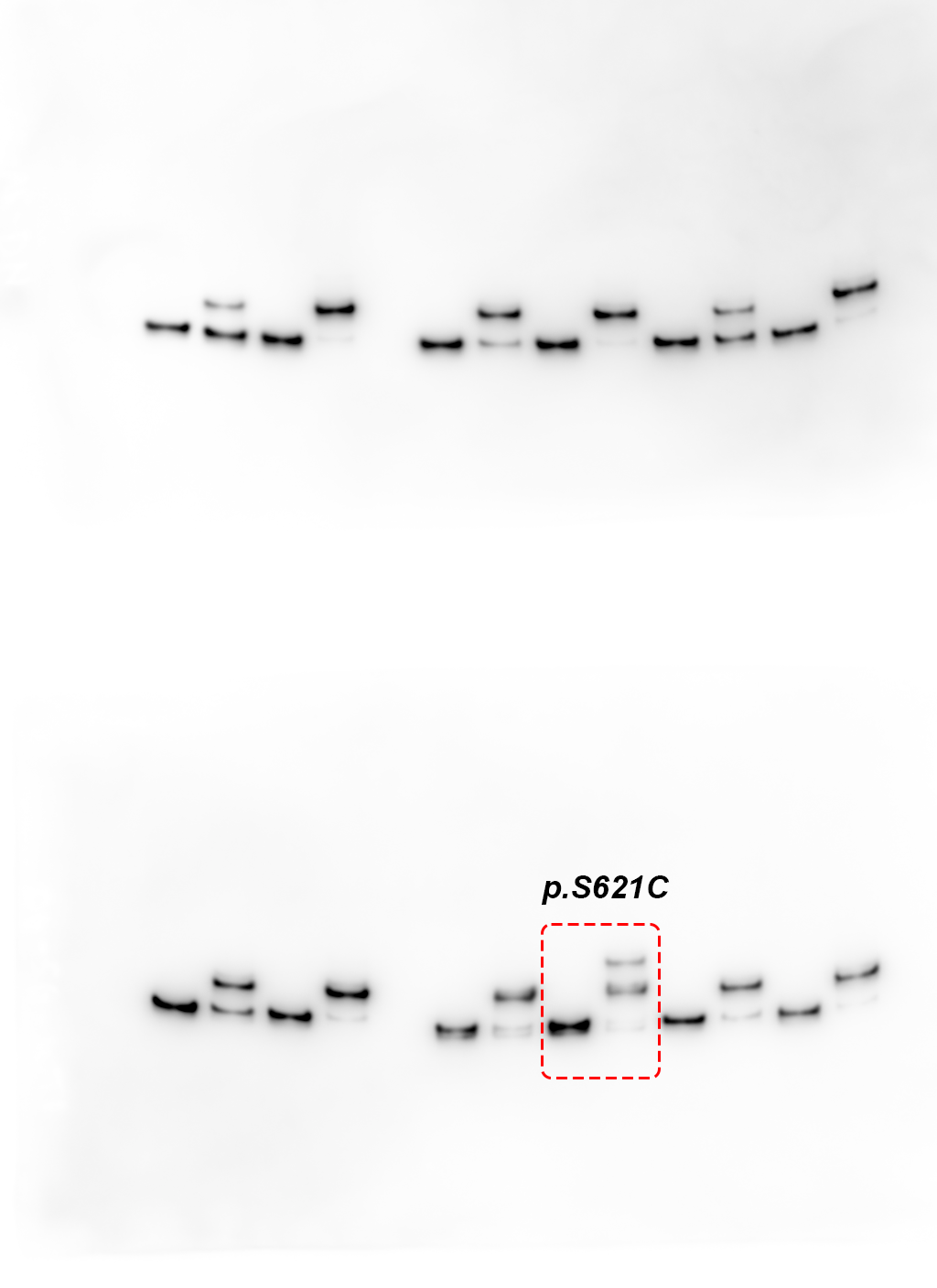

Supplement: Supplementary file 6 — Source Data Fig. 3 [file 44318_2024_46_MOESM6_ESM.zip › EMBOJ-2023-115688_Figure 3/3C/EMBOJ-2023-115688_SourceDATA_Fig3C_S621C.tif]

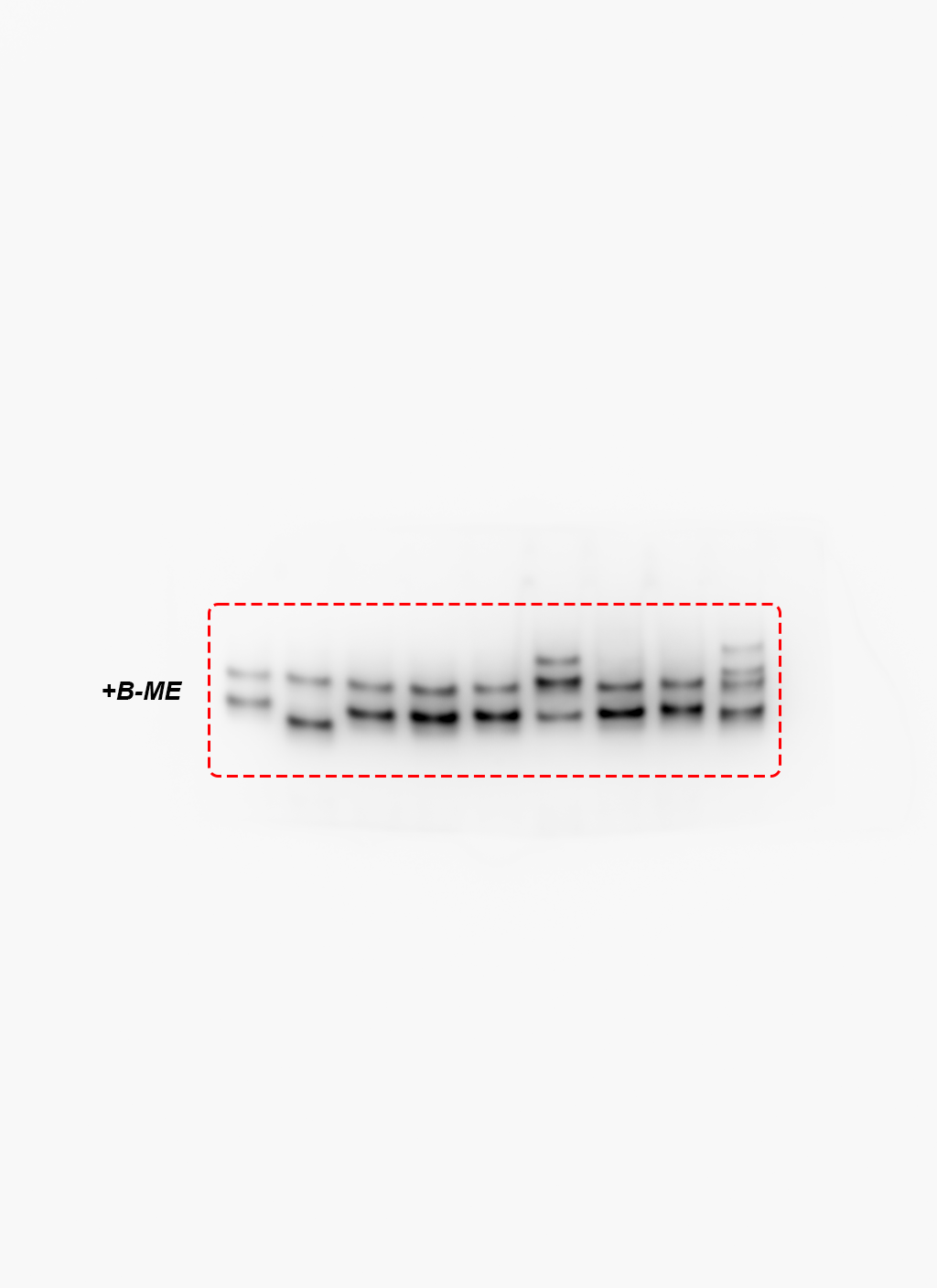

Supplement: Supplementary file 6 — Source Data Fig. 3 [file 44318_2024_46_MOESM6_ESM.zip › EMBOJ-2023-115688_Figure 3/3A/EMBOJ-2023-115688_SourceDATA_Fig3A_BME.tif]

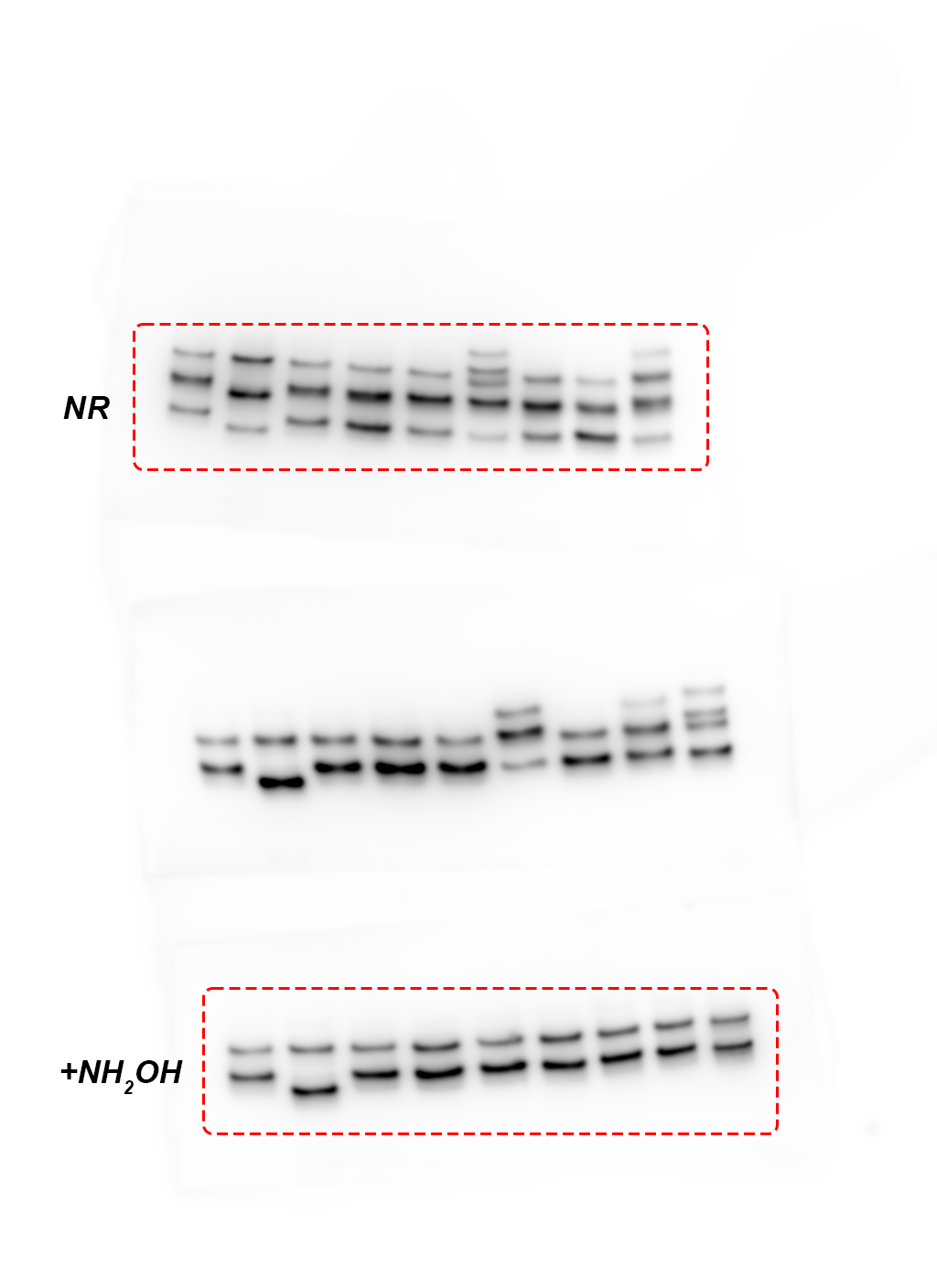

Supplement: Supplementary file 6 — Source Data Fig. 3 [file 44318_2024_46_MOESM6_ESM.zip › EMBOJ-2023-115688_Figure 3/3A/EMBOJ-2023-115688_SourceDATA_Fig3A_NR-Hydroxylamine.tif]

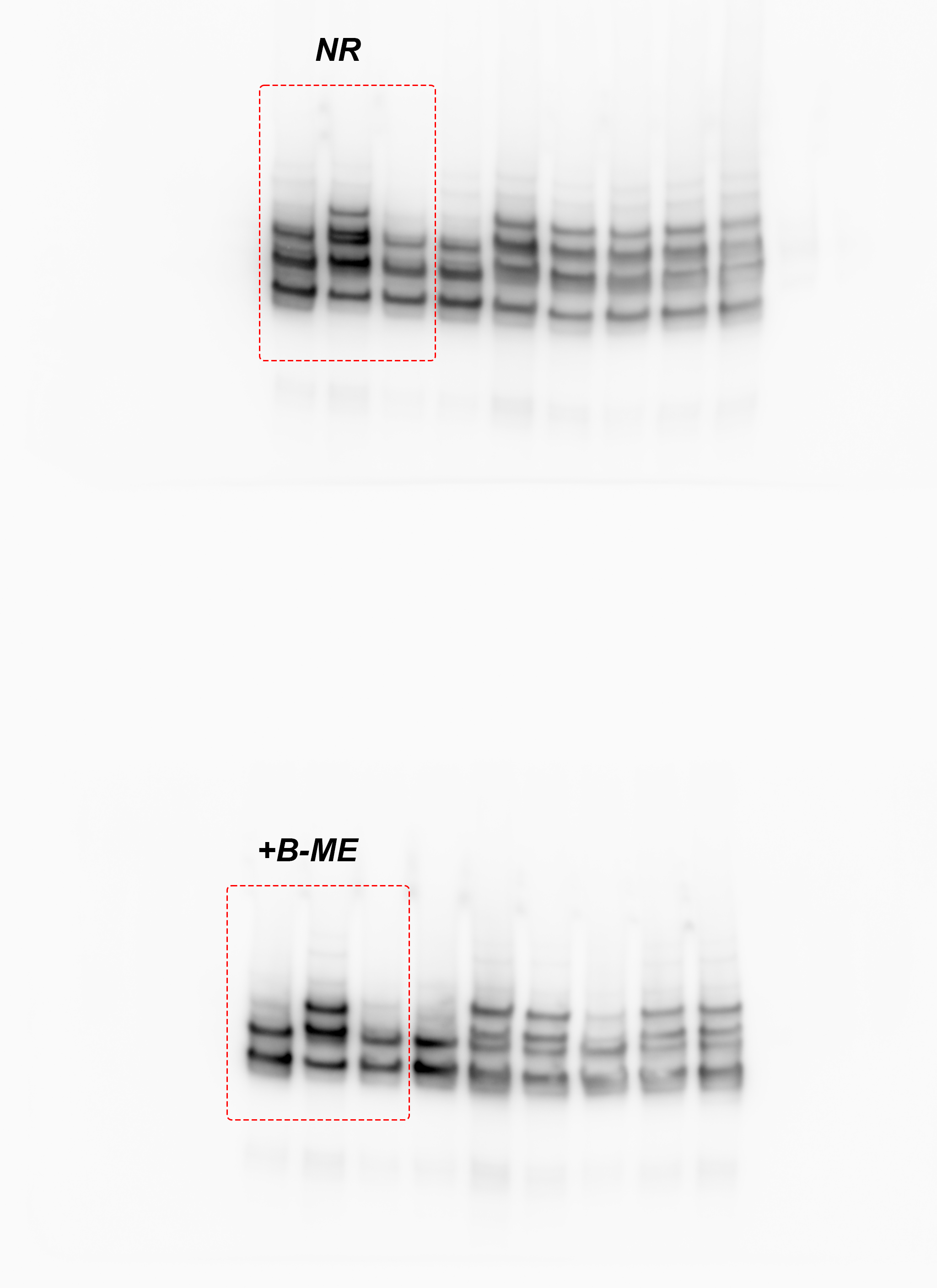

Supplement: Supplementary file 6 — Source Data Fig. 3 [file 44318_2024_46_MOESM6_ESM.zip › EMBOJ-2023-115688_Figure 3/3F/EMBOJ-2023-115688_SourceDATA_Fig3F_NR-BME.tif.tif]

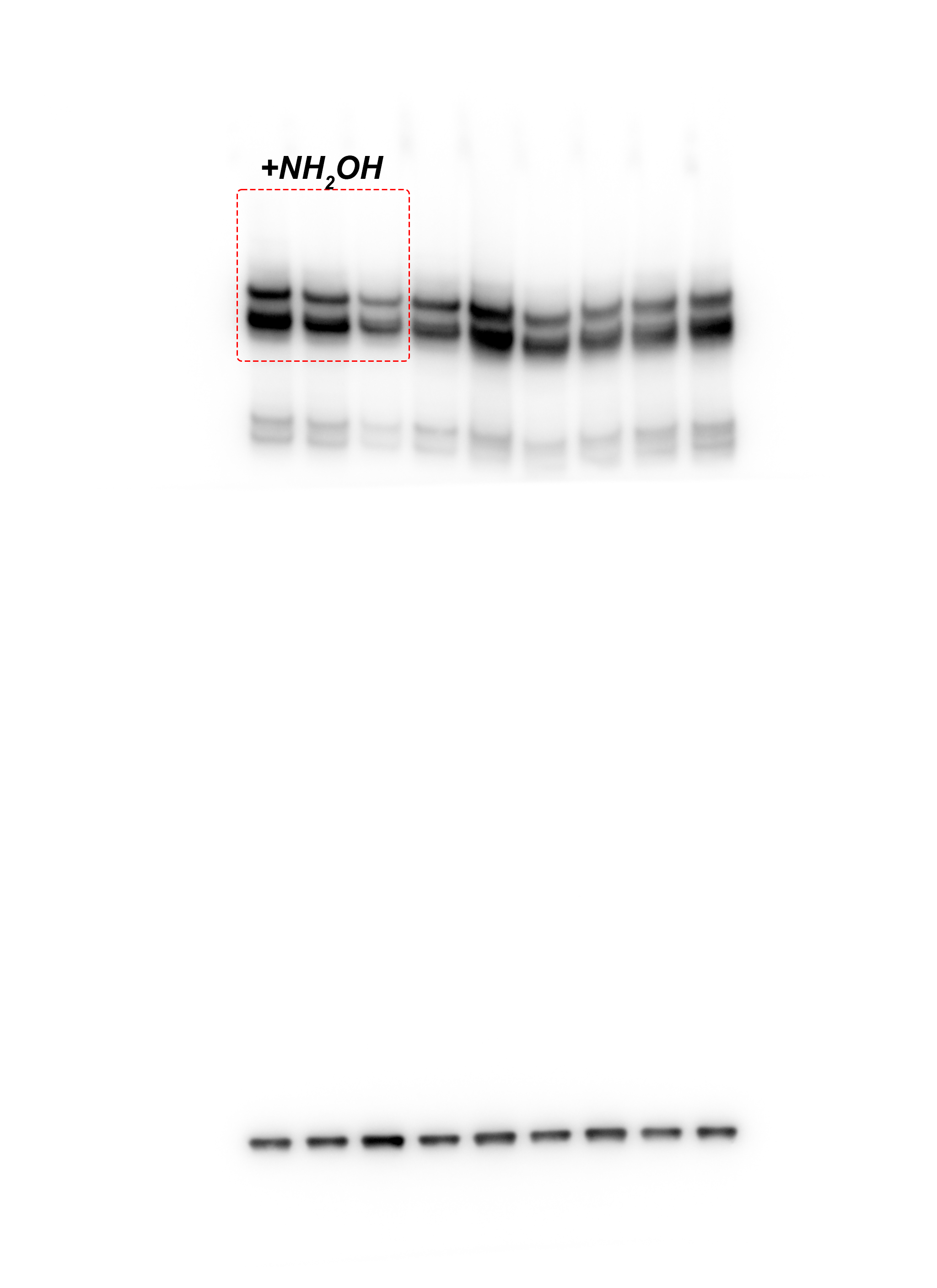

Supplement: Supplementary file 6 — Source Data Fig. 3 [file 44318_2024_46_MOESM6_ESM.zip › EMBOJ-2023-115688_Figure 3/3F/EMBOJ-2023-115688_SourceDATA_Fig3F_Hydroxylamine.tif]

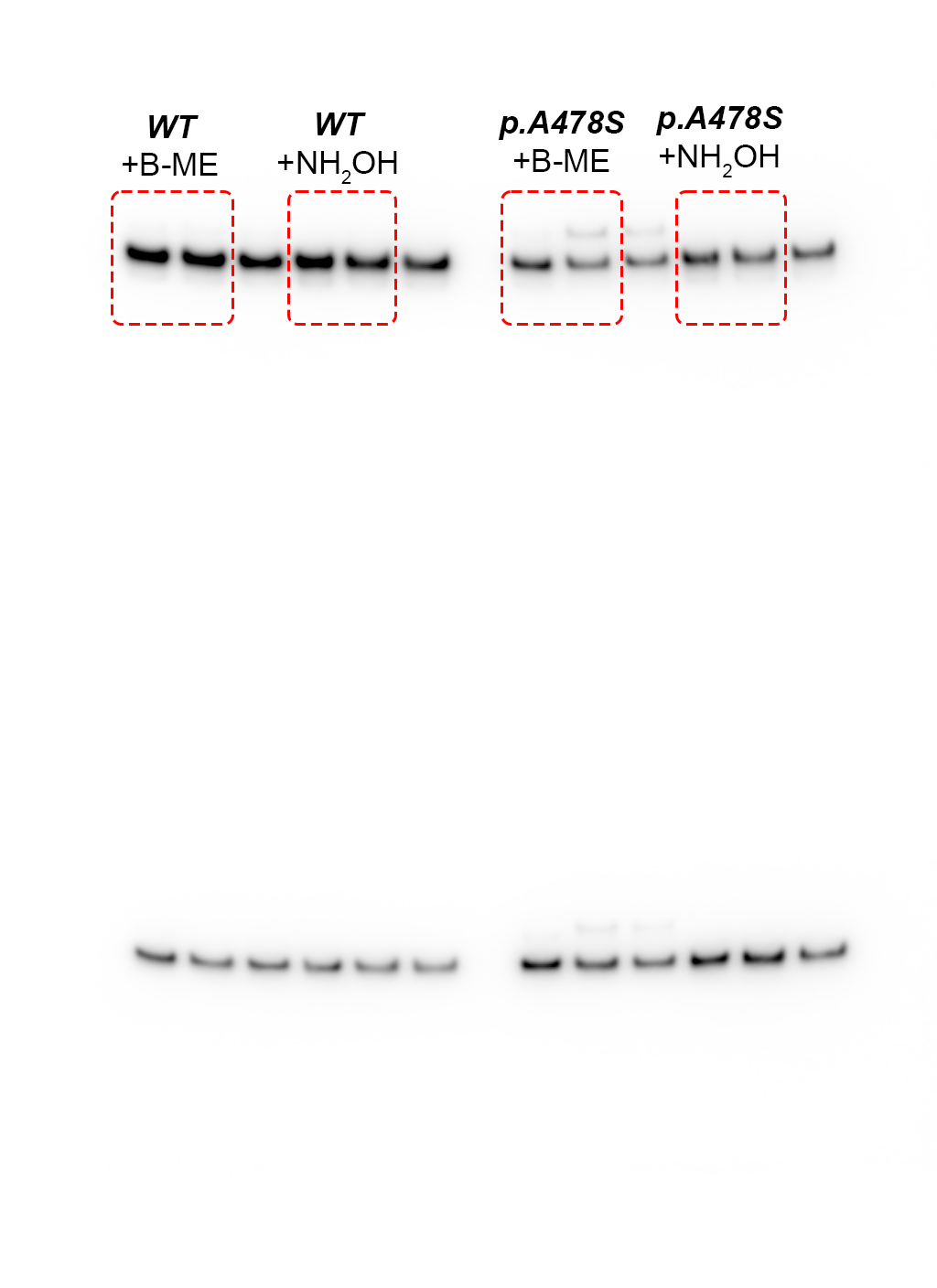

Supplement: Supplementary file 6 — Source Data Fig. 3 [file 44318_2024_46_MOESM6_ESM.zip › EMBOJ-2023-115688_Figure 3/3G/EMBOJ-2023-115688_SourceDATA_Fig3G_BME-Hydroxylamine.tif]

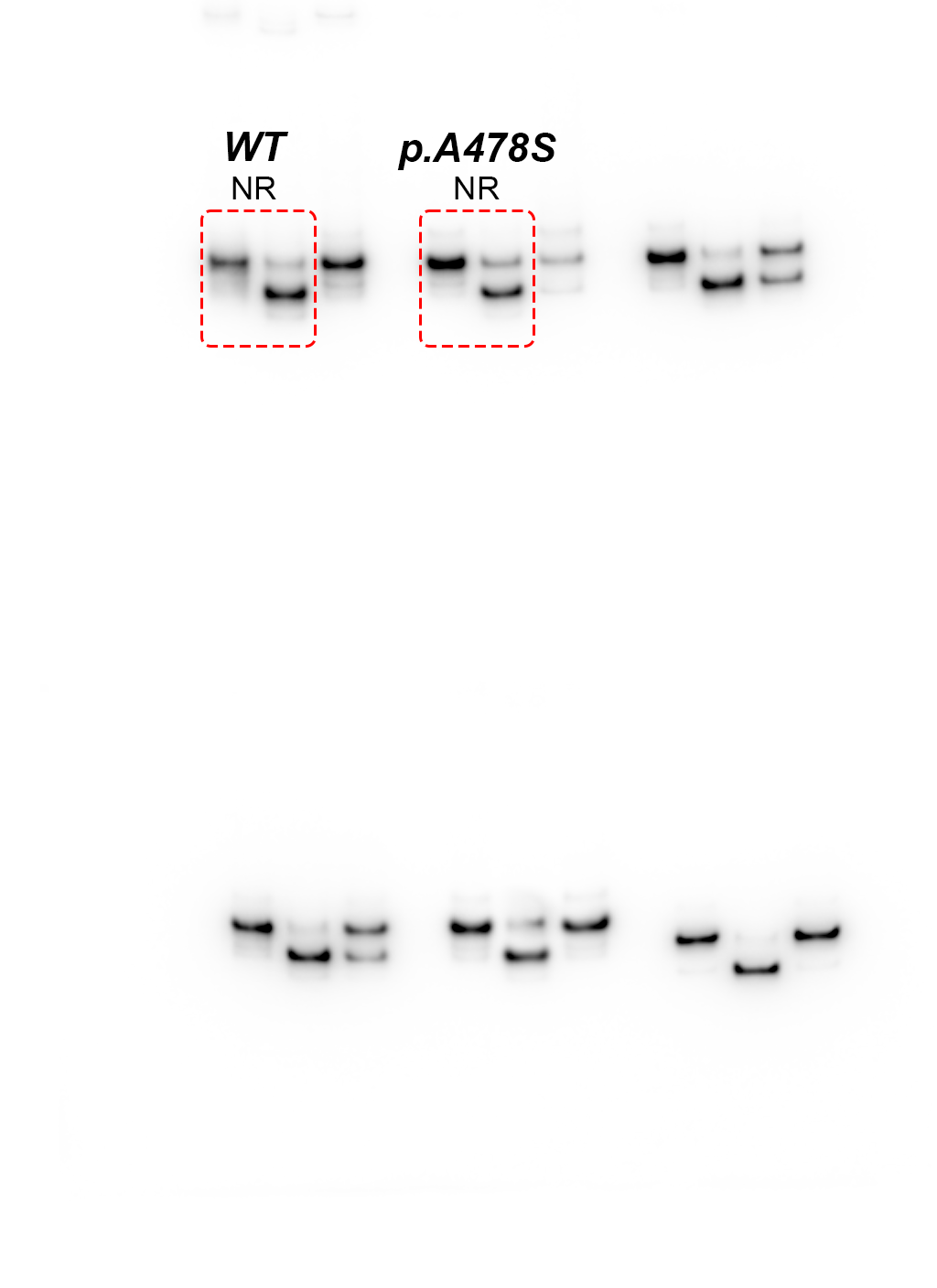

Supplement: Supplementary file 6 — Source Data Fig. 3 [file 44318_2024_46_MOESM6_ESM.zip › EMBOJ-2023-115688_Figure 3/3G/EMBOJ-2023-115688_SourceDATA_Fig3G_NR.tif]

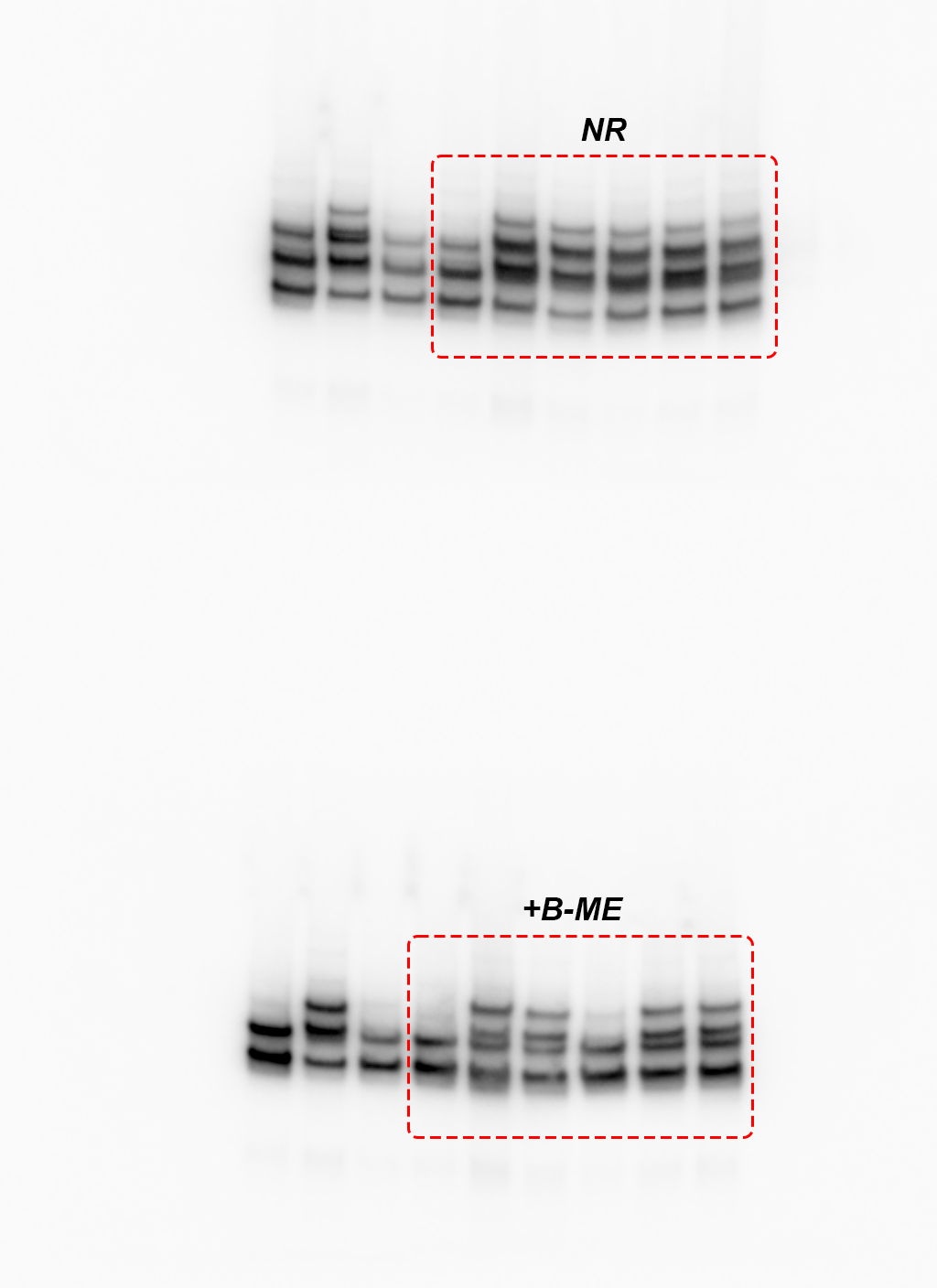

Supplement: Supplementary file 7 — Source Data Fig. 4 [file 44318_2024_46_MOESM7_ESM.zip › EMBOJ-2023-115688_Figure 4/4C/115688_SourceDATA_Fig4C_NR-BME.tif.tif]

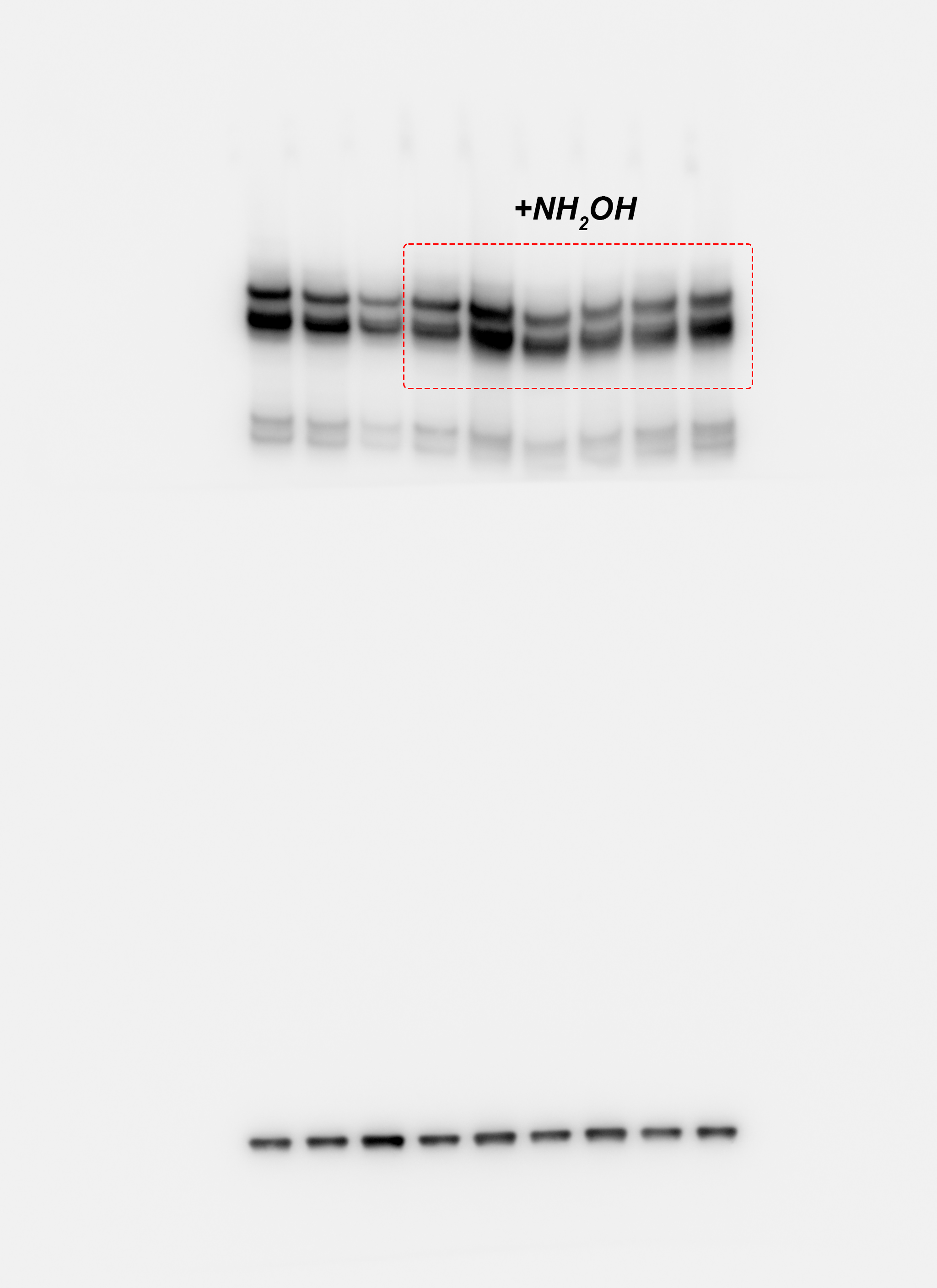

Supplement: Supplementary file 7 — Source Data Fig. 4 [file 44318_2024_46_MOESM7_ESM.zip › EMBOJ-2023-115688_Figure 4/4C/115688_SourceDATA_Fig4C_Hydroxylamine.tif]

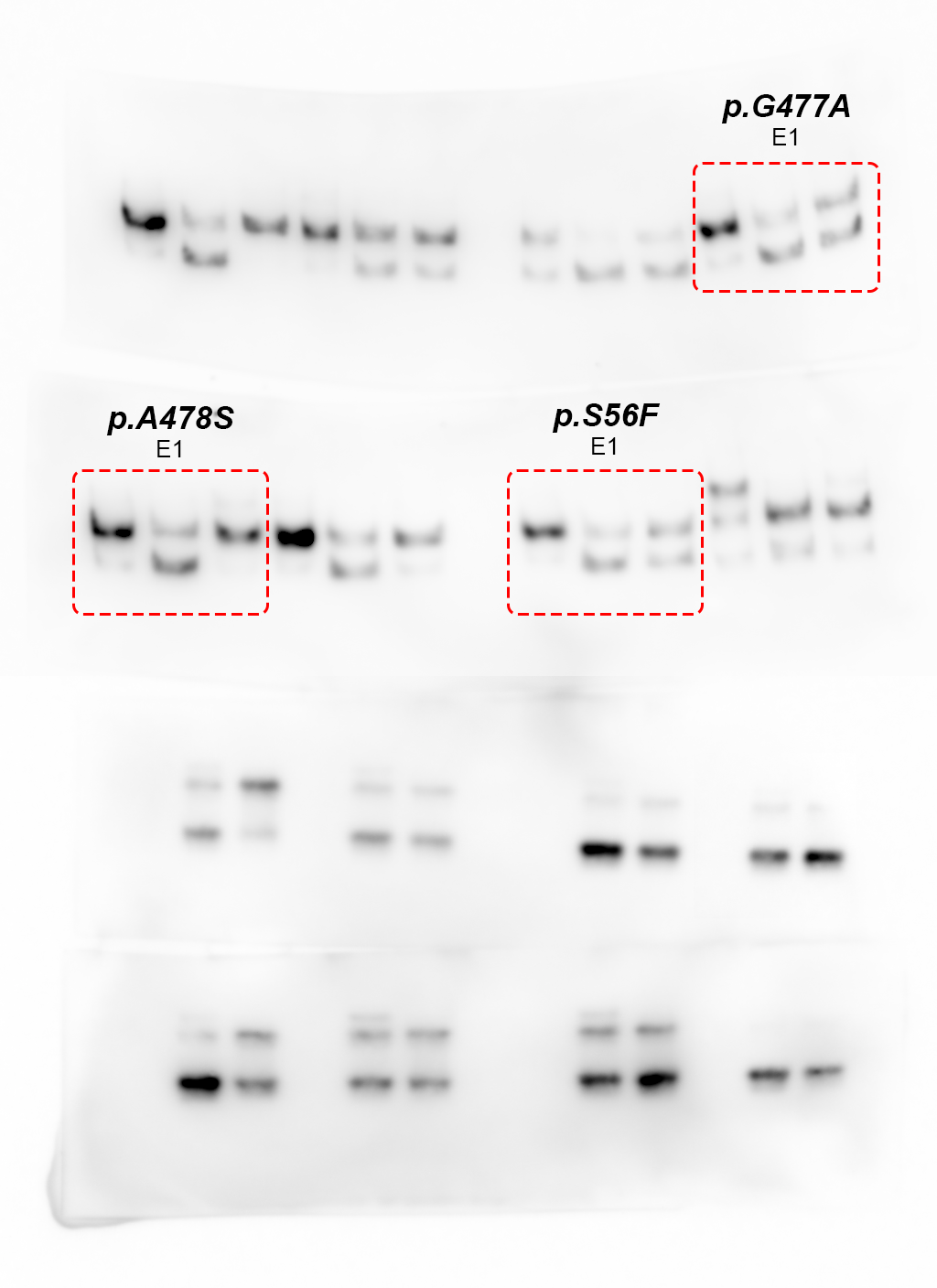

Supplement: Supplementary file 8 — Source Data Fig. 5 [file 44318_2024_46_MOESM8_ESM.zip › EMBOJ-2023-115688_Figure 5/5B/115688_SourceDATA_Fig5B_E1_G477A-A478S-S56F.tif]

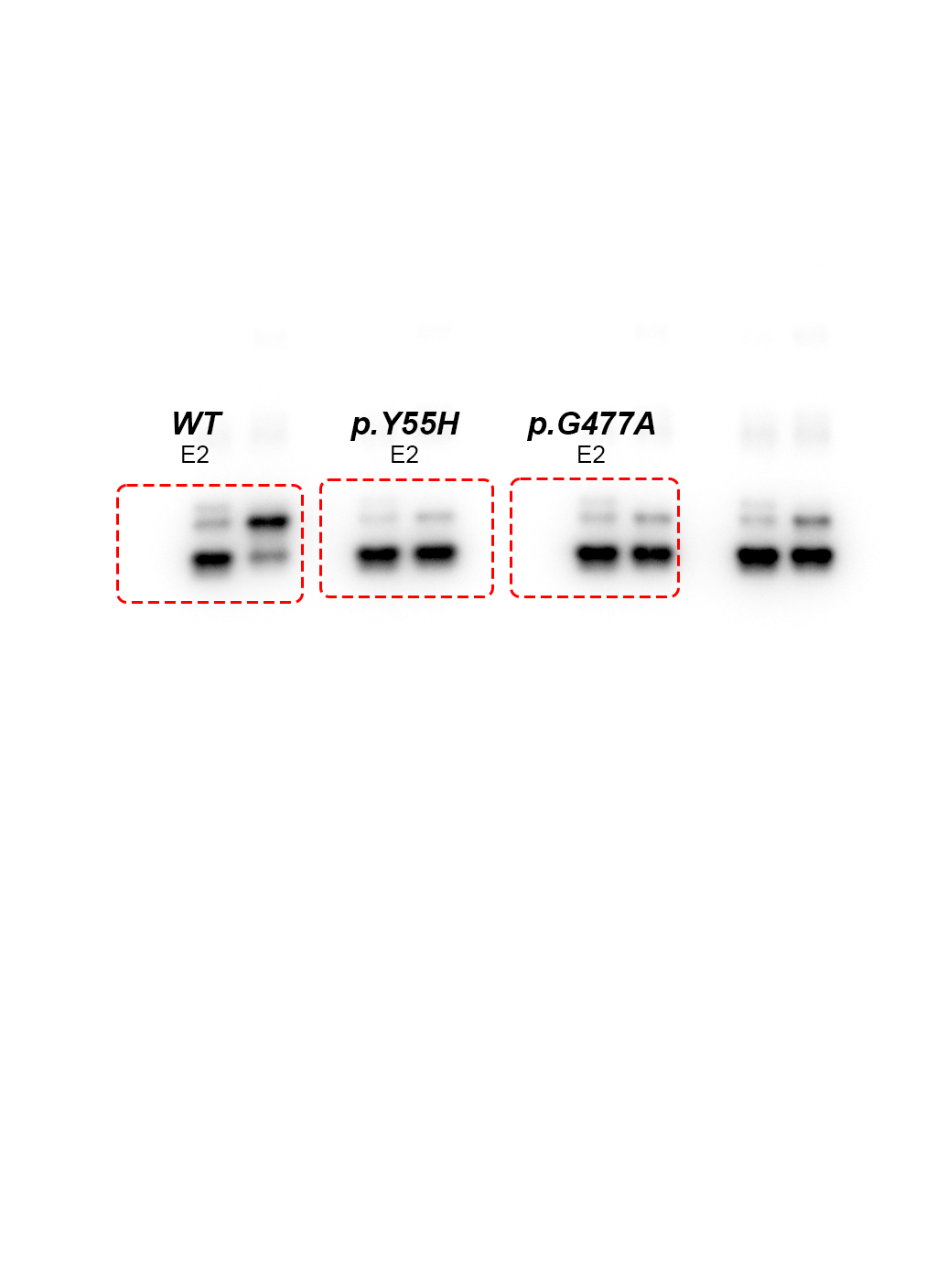

Supplement: Supplementary file 8 — Source Data Fig. 5 [file 44318_2024_46_MOESM8_ESM.zip › EMBOJ-2023-115688_Figure 5/5B/115688_SourceDATA_Fig5B_E2_WT-Y55H-G477A.tif]

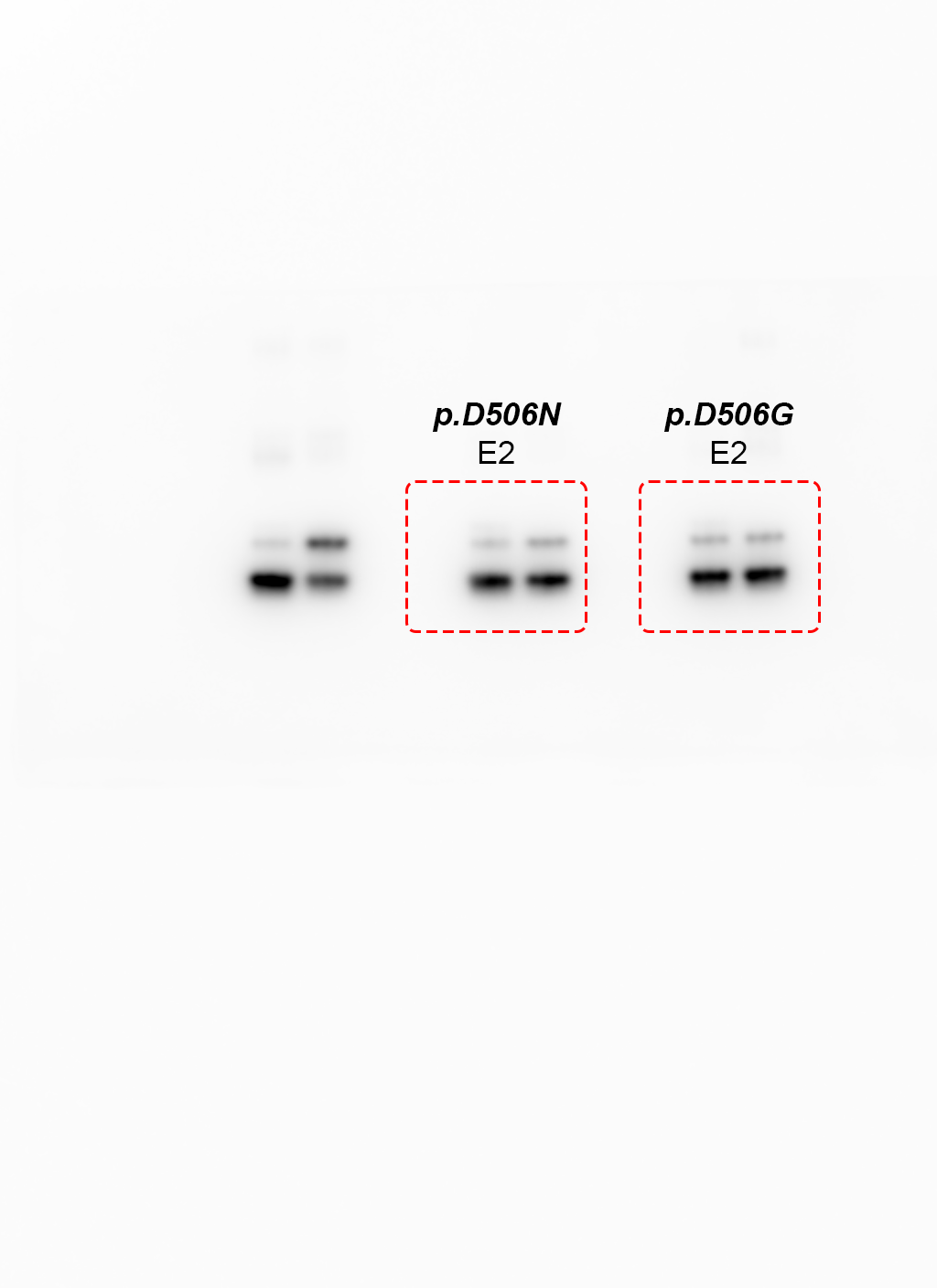

Supplement: Supplementary file 8 — Source Data Fig. 5 [file 44318_2024_46_MOESM8_ESM.zip › EMBOJ-2023-115688_Figure 5/5B/115688_SourceDATA_Fig5B_E2_D506N_D506G.tif]

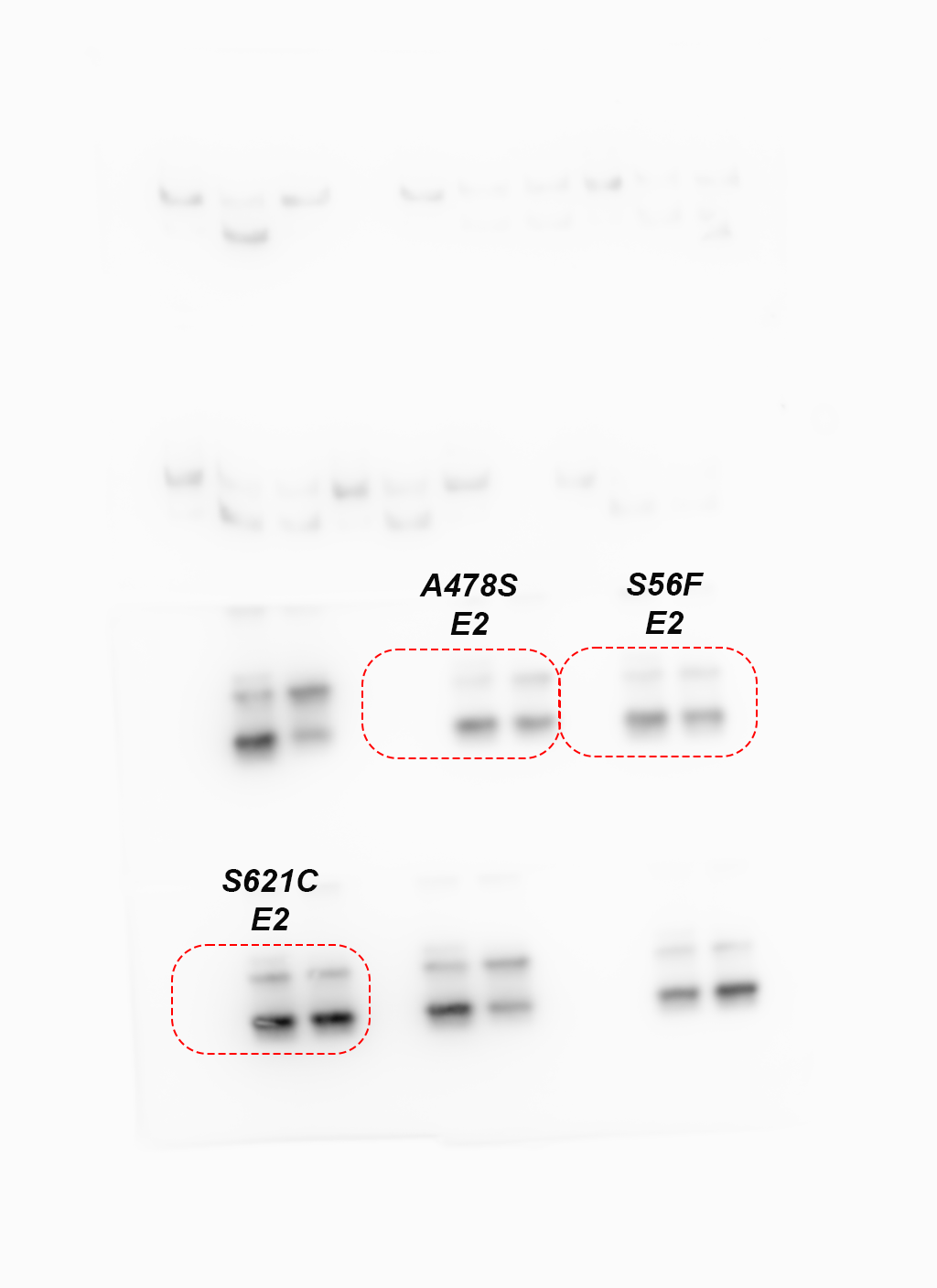

Supplement: Supplementary file 8 — Source Data Fig. 5 [file 44318_2024_46_MOESM8_ESM.zip › EMBOJ-2023-115688_Figure 5/5B/115688_SourceDATA_Fig5B_E2_S56F-A478S-S621C.tif]

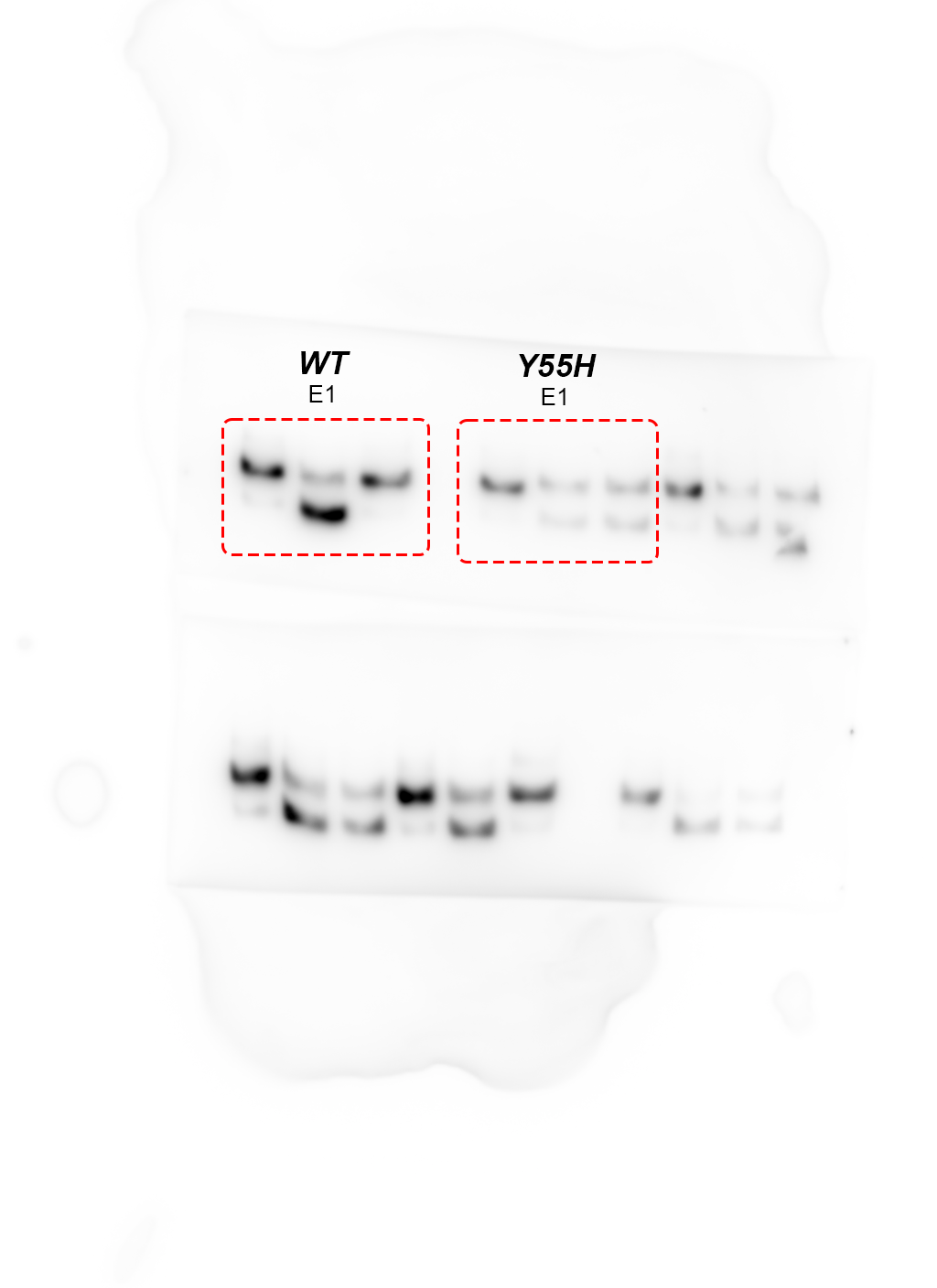

Supplement: Supplementary file 8 — Source Data Fig. 5 [file 44318_2024_46_MOESM8_ESM.zip › EMBOJ-2023-115688_Figure 5/5B/115688_SourceDATA_Fig5B_E1_WT-Y55H.tif]

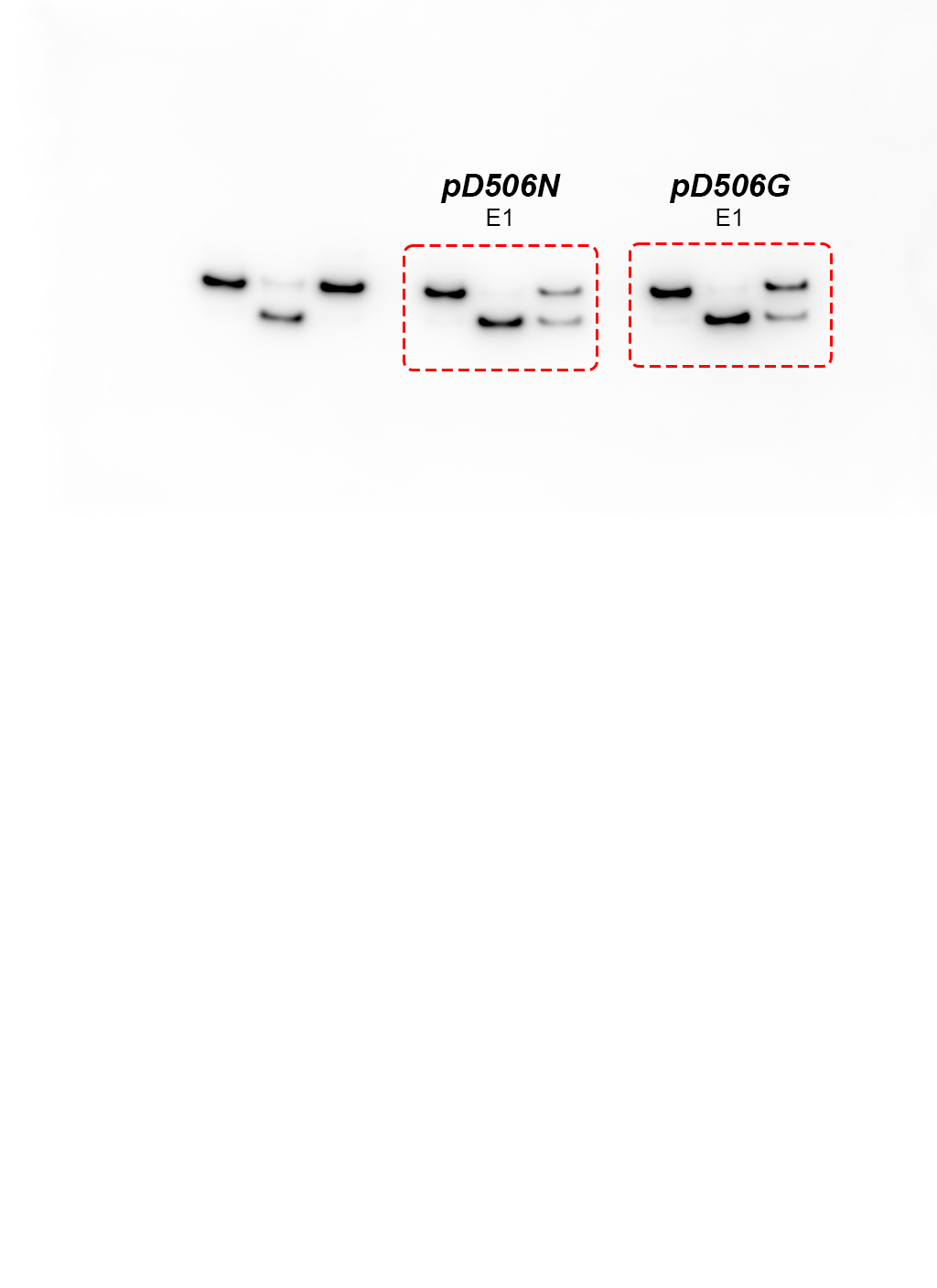

Supplement: Supplementary file 8 — Source Data Fig. 5 [file 44318_2024_46_MOESM8_ESM.zip › EMBOJ-2023-115688_Figure 5/5B/115688_SourceDATA_Fig5B_E1_D506N-D506G.tif]

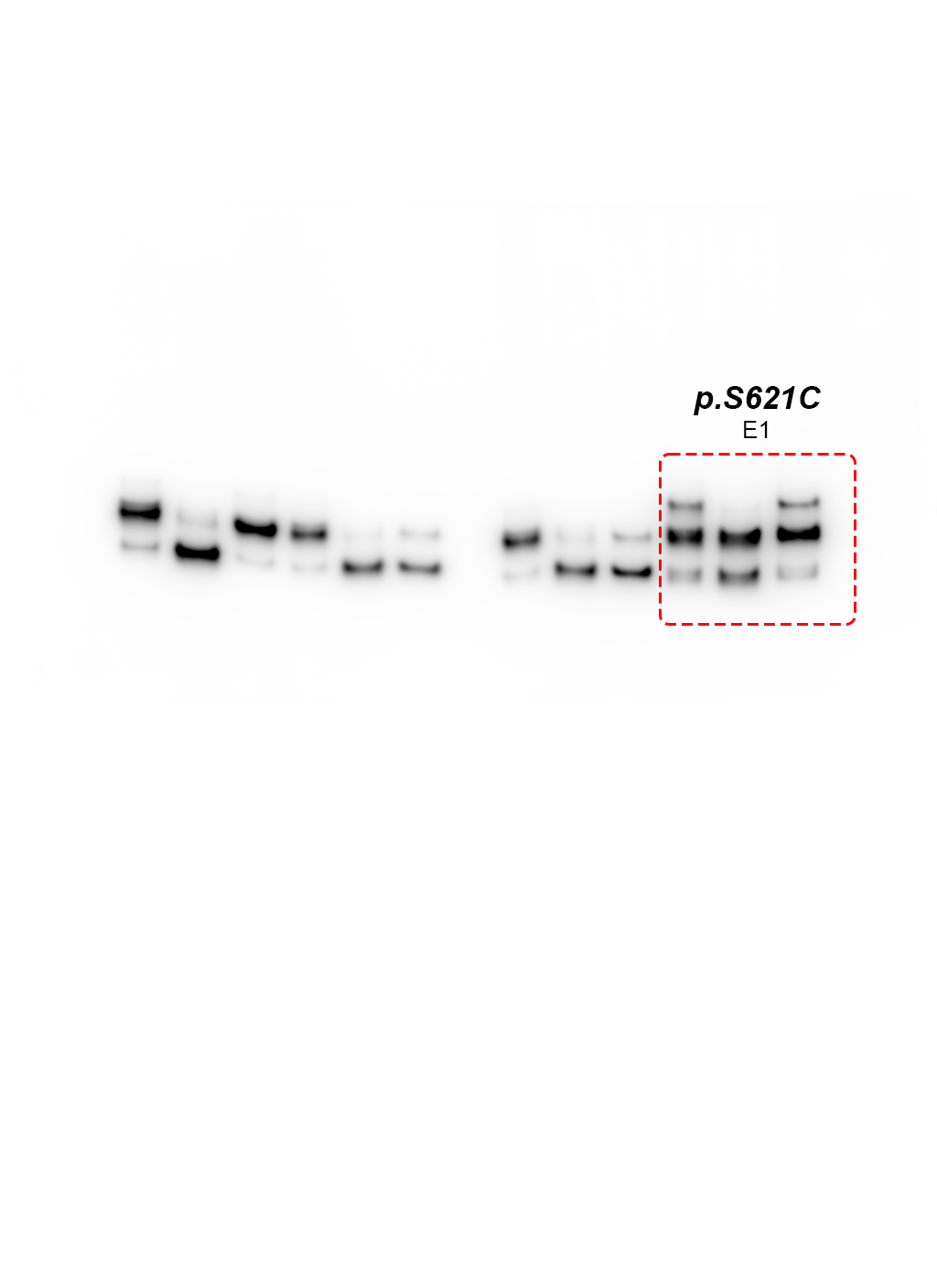

Supplement: Supplementary file 8 — Source Data Fig. 5 [file 44318_2024_46_MOESM8_ESM.zip › EMBOJ-2023-115688_Figure 5/5B/115688_SourceDATA_Fig5B_E1_S621C.tif]

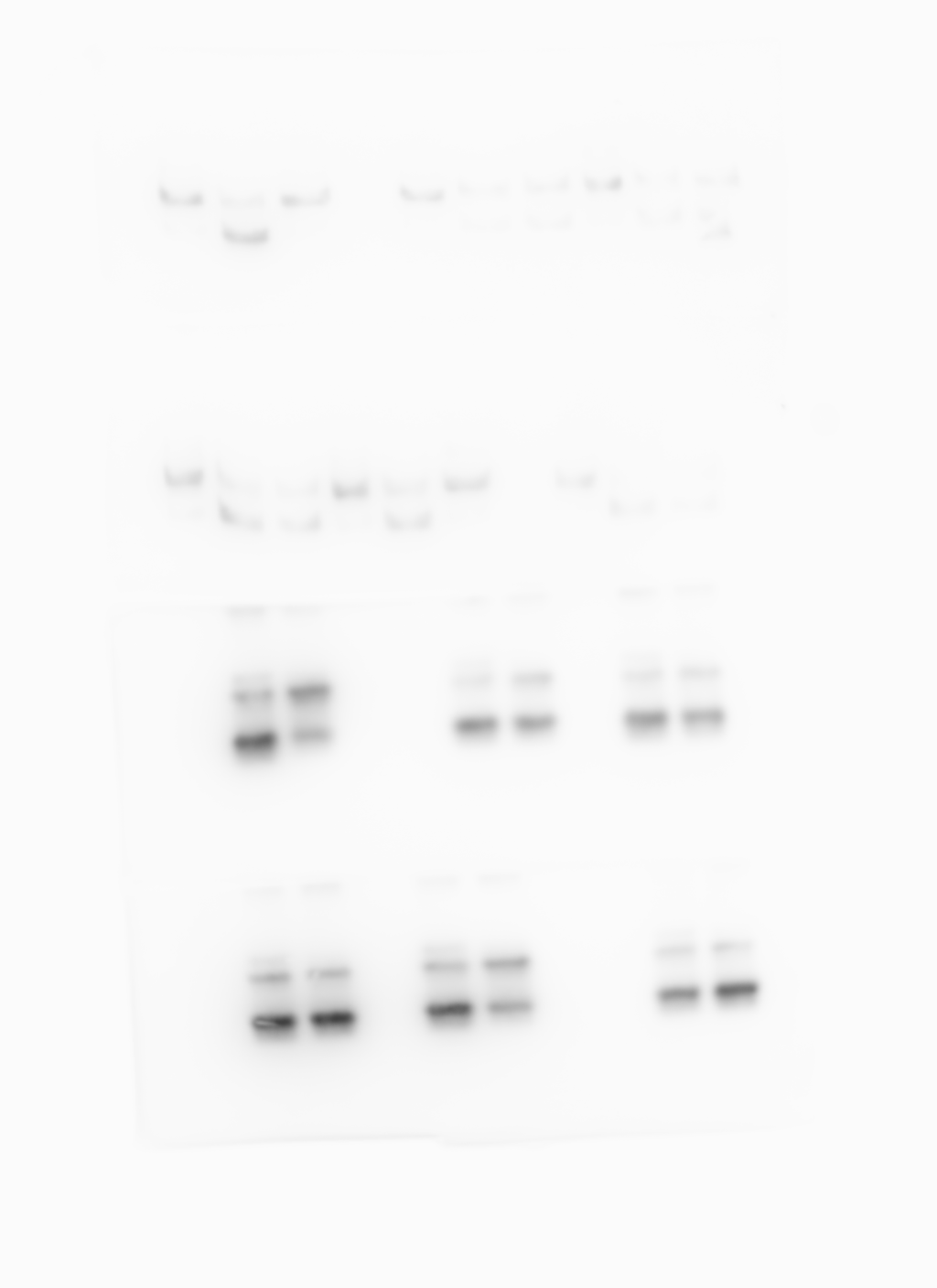

Supplement: Supplementary file 8 — Source Data Fig. 5 [file 44318_2024_46_MOESM8_ESM.zip › EMBOJ-2023-115688_Figure 5/5B/06172023_E2tx-VEXAS-60minPh3 2023.08.17_08.43.28-01_Ch.tif]

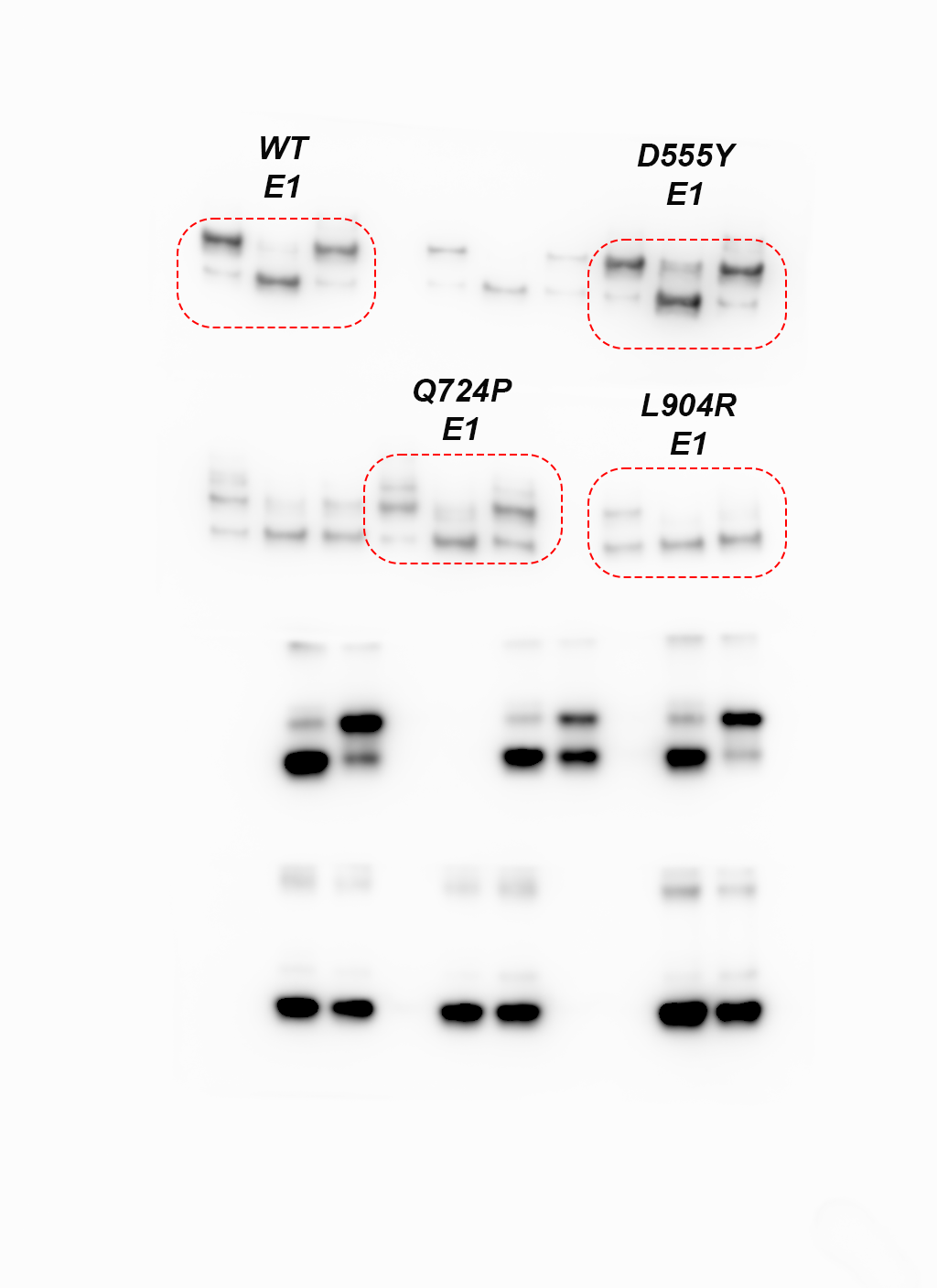

Supplement: Supplementary file 10 — Source Data Fig. 7 [file 44318_2024_46_MOESM10_ESM.zip › EMBOJ-2023-115688_Figure 7/7E/115688_SourceDATA_Fig7E_E1_WT-D555Y-Q724P-L904R.tif]

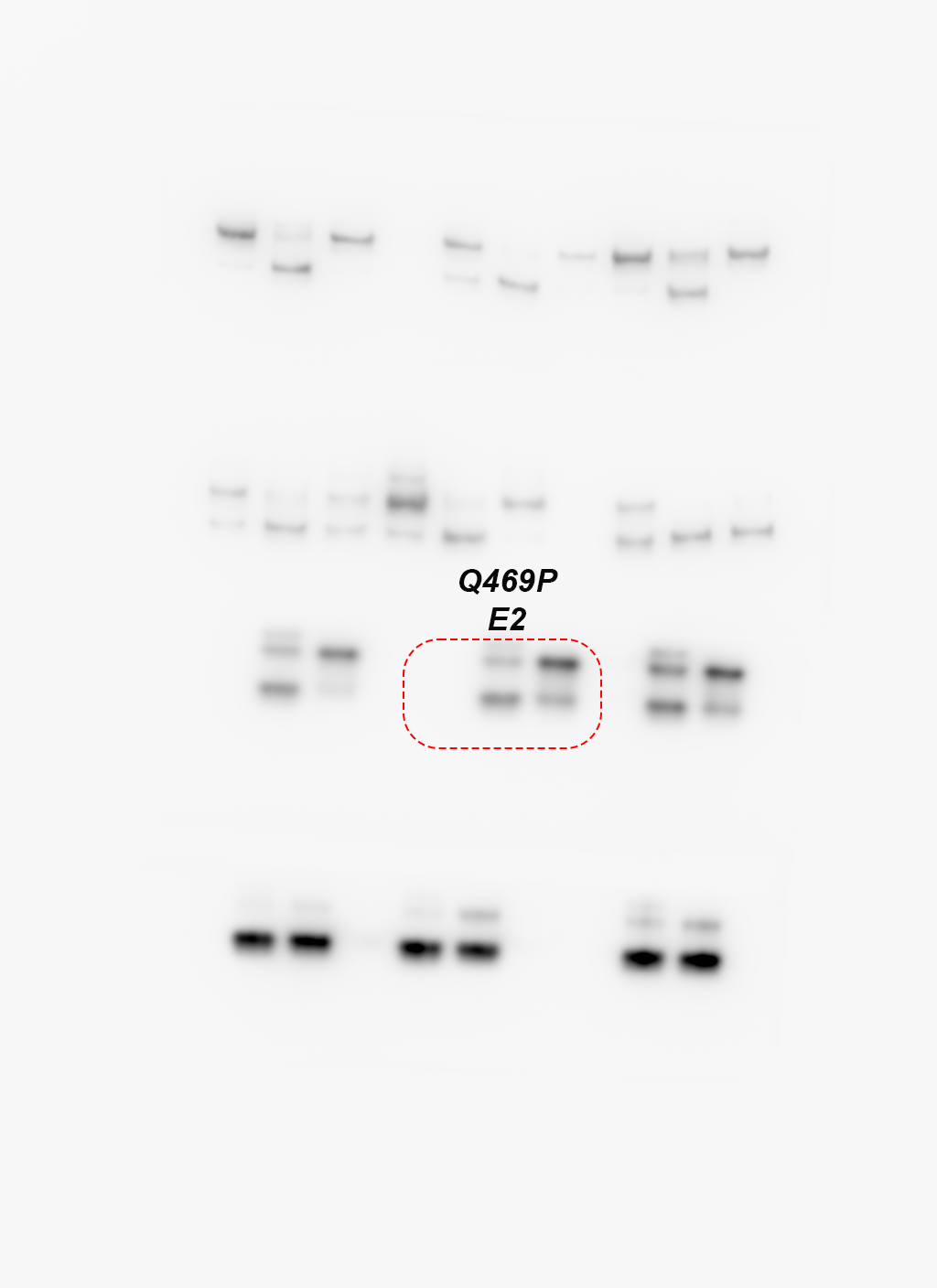

Supplement: Supplementary file 10 — Source Data Fig. 7 [file 44318_2024_46_MOESM10_ESM.zip › EMBOJ-2023-115688_Figure 7/7E/115688_SourceDATA_Fig7E_E2_Q469P.tif]

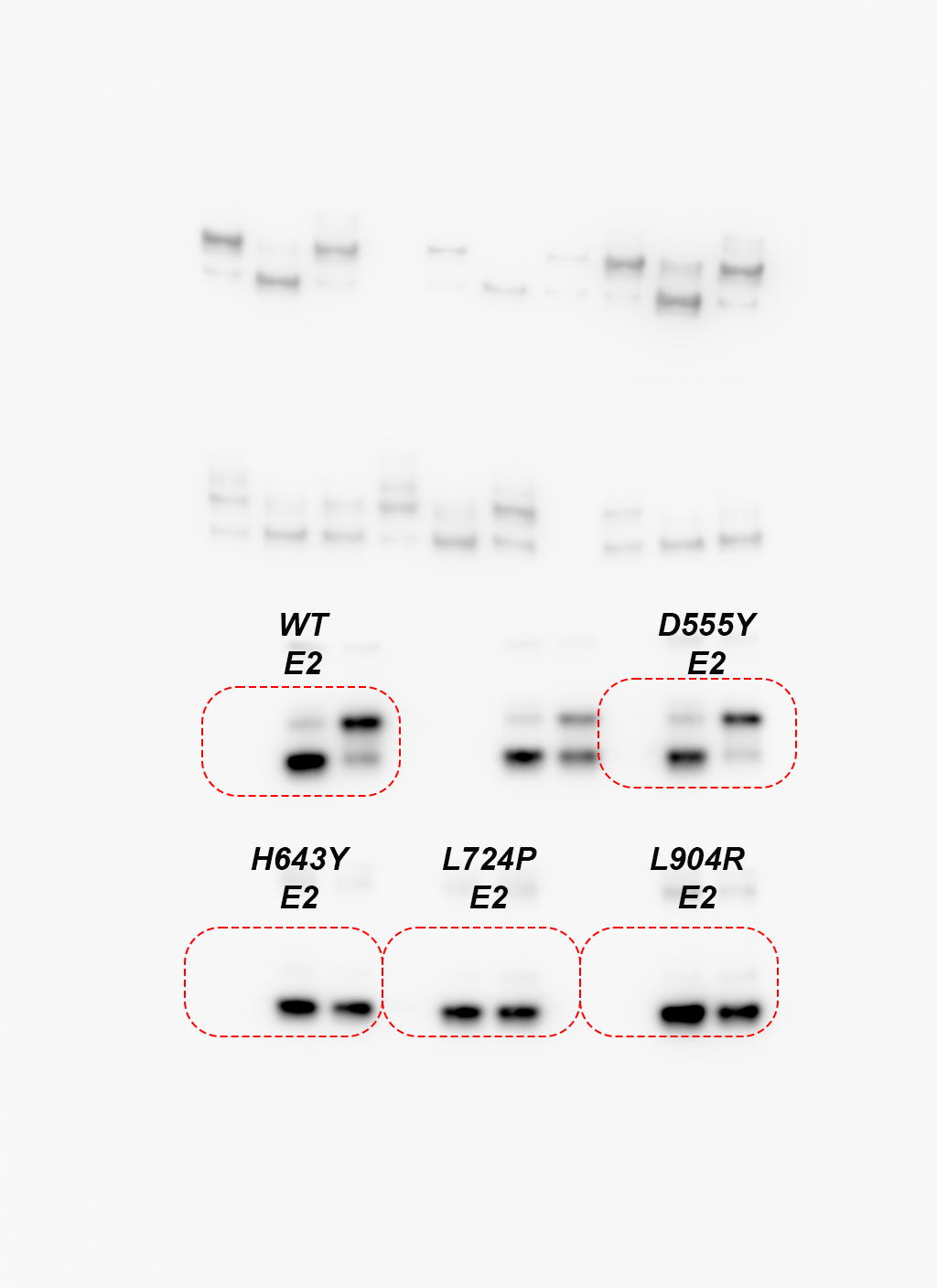

Supplement: Supplementary file 10 — Source Data Fig. 7 [file 44318_2024_46_MOESM10_ESM.zip › EMBOJ-2023-115688_Figure 7/7E/115688_SourceDATA_Fig7E_E2_WT-D555Y-H643Y-Q724P-L904R.tif]

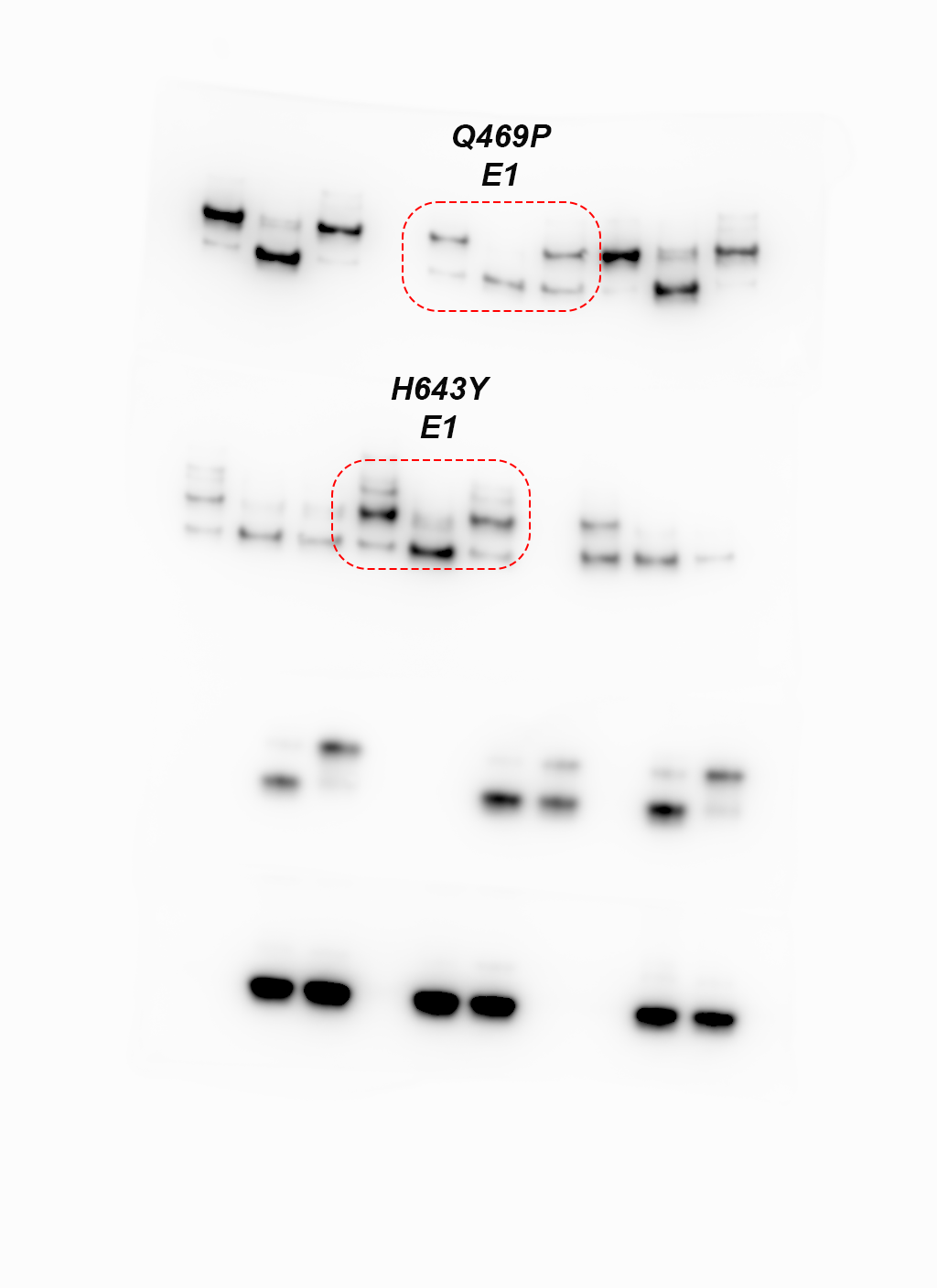

Supplement: Supplementary file 10 — Source Data Fig. 7 [file 44318_2024_46_MOESM10_ESM.zip › EMBOJ-2023-115688_Figure 7/7E/115688_SourceDATA_Fig7E_E1_Q469P_H643Y.tif]

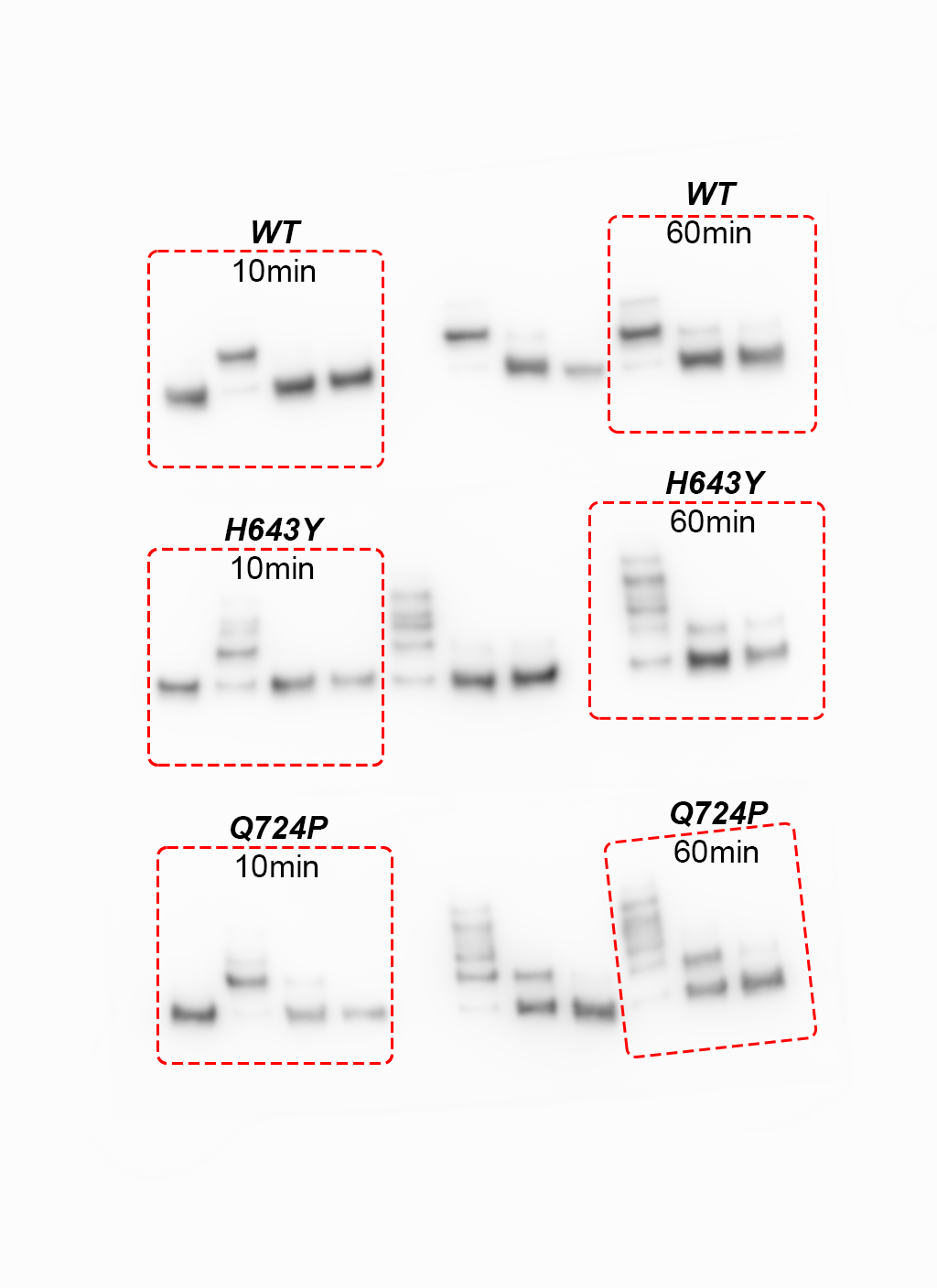

Supplement: Supplementary file 10 — Source Data Fig. 7 [file 44318_2024_46_MOESM10_ESM.zip › EMBOJ-2023-115688_Figure 7/7A/115688_SourceDATA_Fig7A_Thioester Formation Assay.tif]
