# Supplementary material for: Shared and distinct mechanisms of UBA1 inactivation across different diseases
Source: EMBO J. 2024 Feb 15;43(10):1919–46. doi: 10.1038/s44318-024-00046-z (PMC11099125; doi:10.1038/s44318-024-00046-z)
Supplement: Supplementary file 13 — Expanded View Figures [file 44318_2024_46_MOESM13_ESM.pdf]

Expanded View Figures

Figure EV1. Identification and clinical phenotypes of novel non-pMet41 VEXAS mutations.

(**A**) Sanger sequencing confirming novel variants. (**B**) Digital droplet PCR (ddPCR) confirmation for novel variants in P3, P4, P5. $n = 3$ technical replicates, error bars = s.d. (**C**) Conservation of protein sequence for UBA1. (**D**) Cytoplasmic vacuoles were seen in a subset of the proerythroblasts (Panel i, ii, iii) and promyelocytes (Panel iii, iv, v, vi) from P6. Scale bar = 20 µm. (**E**) Sweets syndrome in P6.

This is a U.S. Government work and not under copyright protection in the US; foreign copyright protection may apply

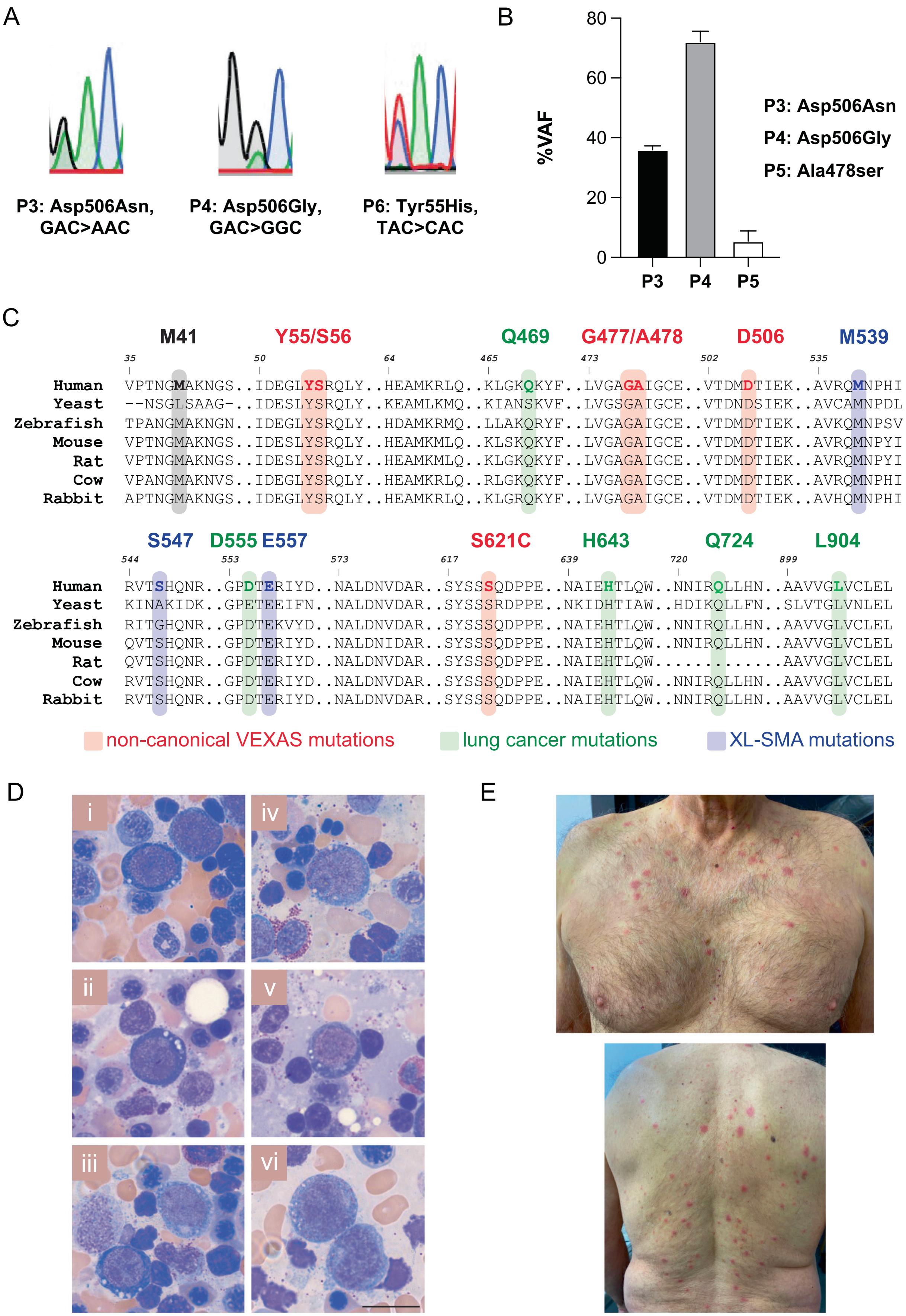

 This is a U.S. Government work and not under copyright protection in the US; foreign copyright protection may apply

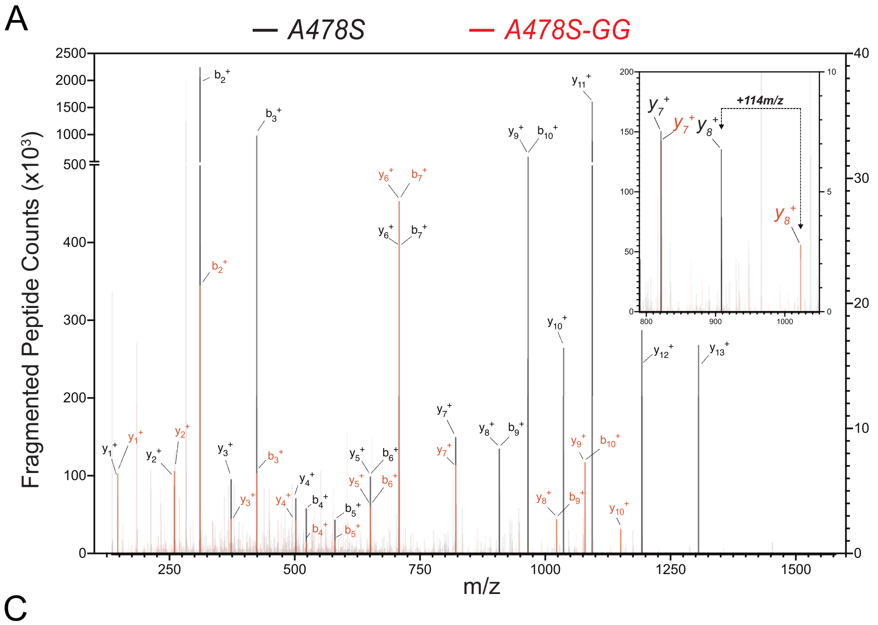

B

	A478S	$A478S_{Oxy}$	Δm/z
y_{13}	1305.69	n/a	n/a
y_{12}	1192.61	1306.65	114.04
y_{11}	1093.54	1207.58	114.04
y_{10}	1036.52	1150.56	114.04
y_9	965.48	1079.52	114.04
y_8	908.46	1022.50	114.04
y_7	821.43	821.43	0.00
y_6	708.34	708.34	0.00
y_5	651.32	651.32	0.00
y_4	502.32	502.32	0.00
y_3	373.28	373.28	0.00
y_2	260.20	260.20	0.00
y_1	147.11	147.11	0.00

Peptide Fragments (1+)

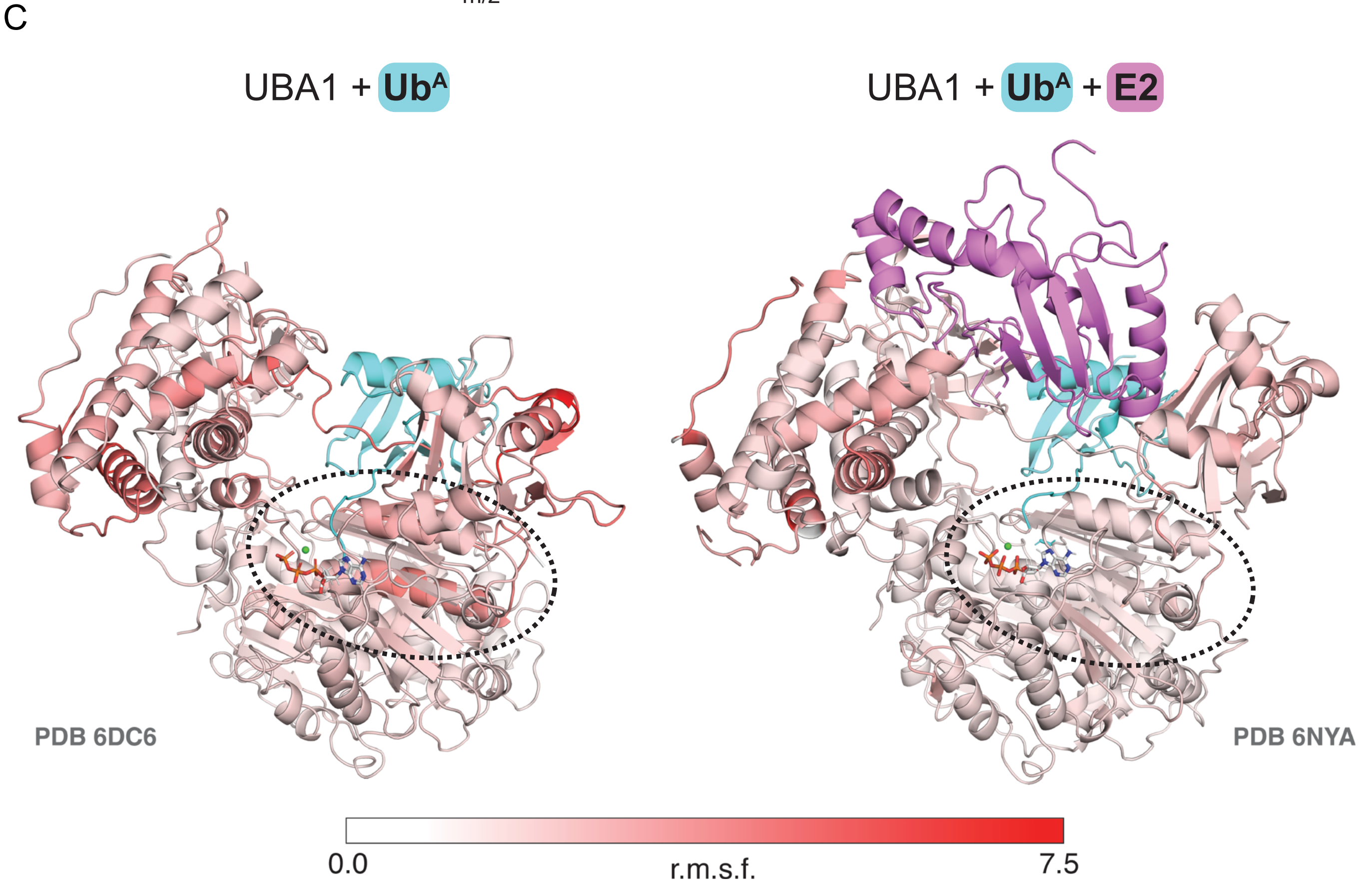

Figure EV2. UBA1 p.A478S forms an aberrant oxyester at the mutation site.

(**A**) Annotated MS/MS spectrum of A478S peptide with (red) and without (black) diGly remnant. Insert highlights the difference of 114 Da, indicative of the diGly remnant. (**B**) Table summarizing the masses of fragments of the unmodified and ubiquitylated A478S peptide, pinpointing the diGly remnant (Δ*m/z* 114.04 Da) on S478. (**C**) Side-by-side ribbon diagrams of UBA1 bound to ubiquitin (cyan) and ATP (indicated as a stick diagram with carbon, oxygen, nitrogen, and phosphorus in gray, red, blue, and orange) following 100 ns of molecular dynamics in the absence (left, PDB: 6DC6) or presence (right, PDB: 6NYA) of E2 enzyme (Ubc3, purple). The per residue r.m.s.f. (root mean square fluctuation) value is indicated as a red gradient (scale bar). A black dashed circle highlights the region where ubiquitin, ATP, and Mg^{2+} (green sphere) bind.

This is a U.S. Government work and not under copyright protection in the US; foreign copyright protection may apply

A

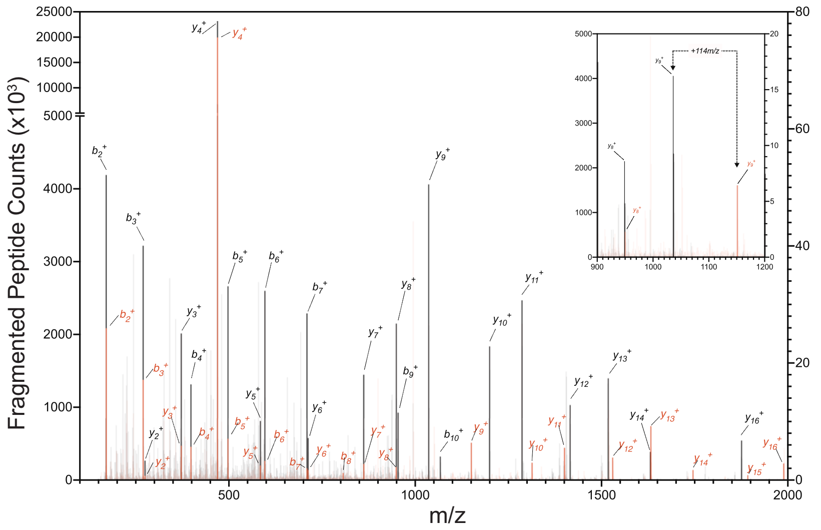

B

Peptide Fragments (1+)

	S621	S621C$_{OxyS619}$	Δm/z
y_{16}	1873.80	1987.84	114.04
y_{15}	n/a	1890.79	n/a
y_{14}	1629.68	1743.72	114.04
y_{13}	1516.59	1630.64	114.04
y_{12}	1415.55	1529.59	114.04
y_{11}	1286.50	1400.55	114.04
y_{10}	1199.47	1313.51	114.04
y_9	1036.41	1150.45	114.04
y_8	949.38	949.38	0.00
y_7	862.34	862.34	0.00
y_6	713.35	713.35	0.00
y_5	585.29	585.29	0.00
y_4	470.26	470.26	0.00
y_3	373.21	373.21	0.00
y_2	276.16	276.16	0.00

C

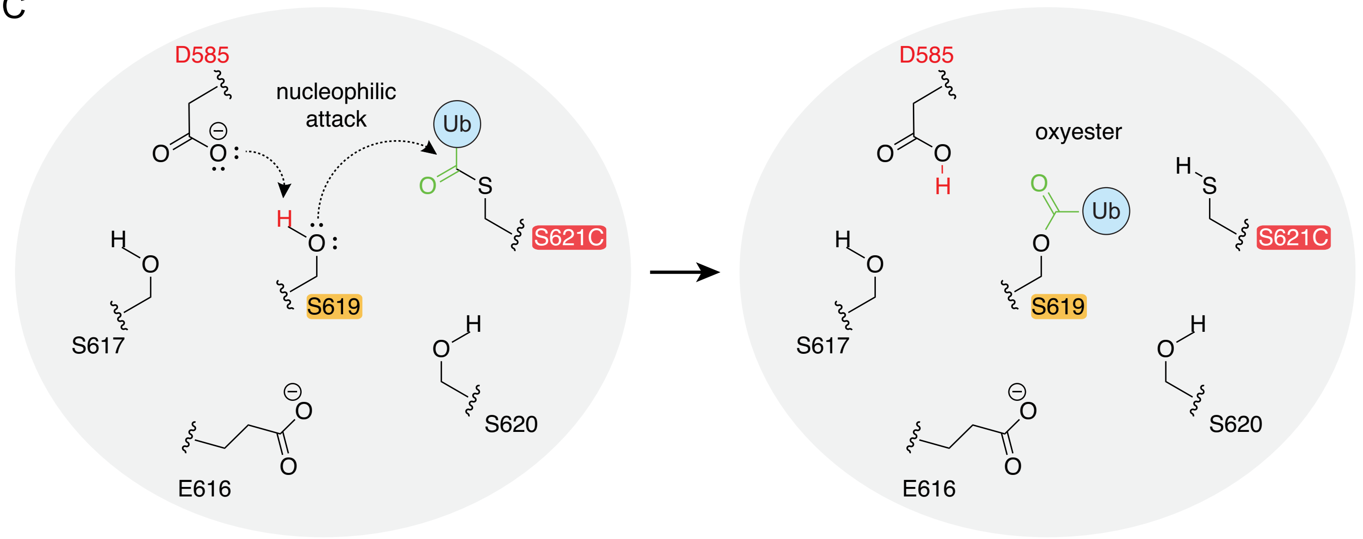

Figure EV3. UBA1 p.S621C forms an aberrant oxyester at S619 via a thioester intermediate at S621C.

(**A**) Annotated MS/MS spectrum of S621C peptide with (red) and without (black) diGly remnant. Insert highlights the difference of 114 Da, indicative of the diGly remnant. (**B**) Table summarizing the masses of fragments of the unmodified and ubiquitylated S621C peptide, pinpointing the diGly remnant (Δ*m/z* 114.04 Da) on S619. (**C**) Proposed catalytic mechanism for oxyester formation through deprotonation of S619 via the putative catalytic base D585 forming an oxyanion that promotes a nucleophilic attack of the thioester at S621C.

 This is a U.S. Government work and not under copyright protection in the US; foreign copyright protection may apply

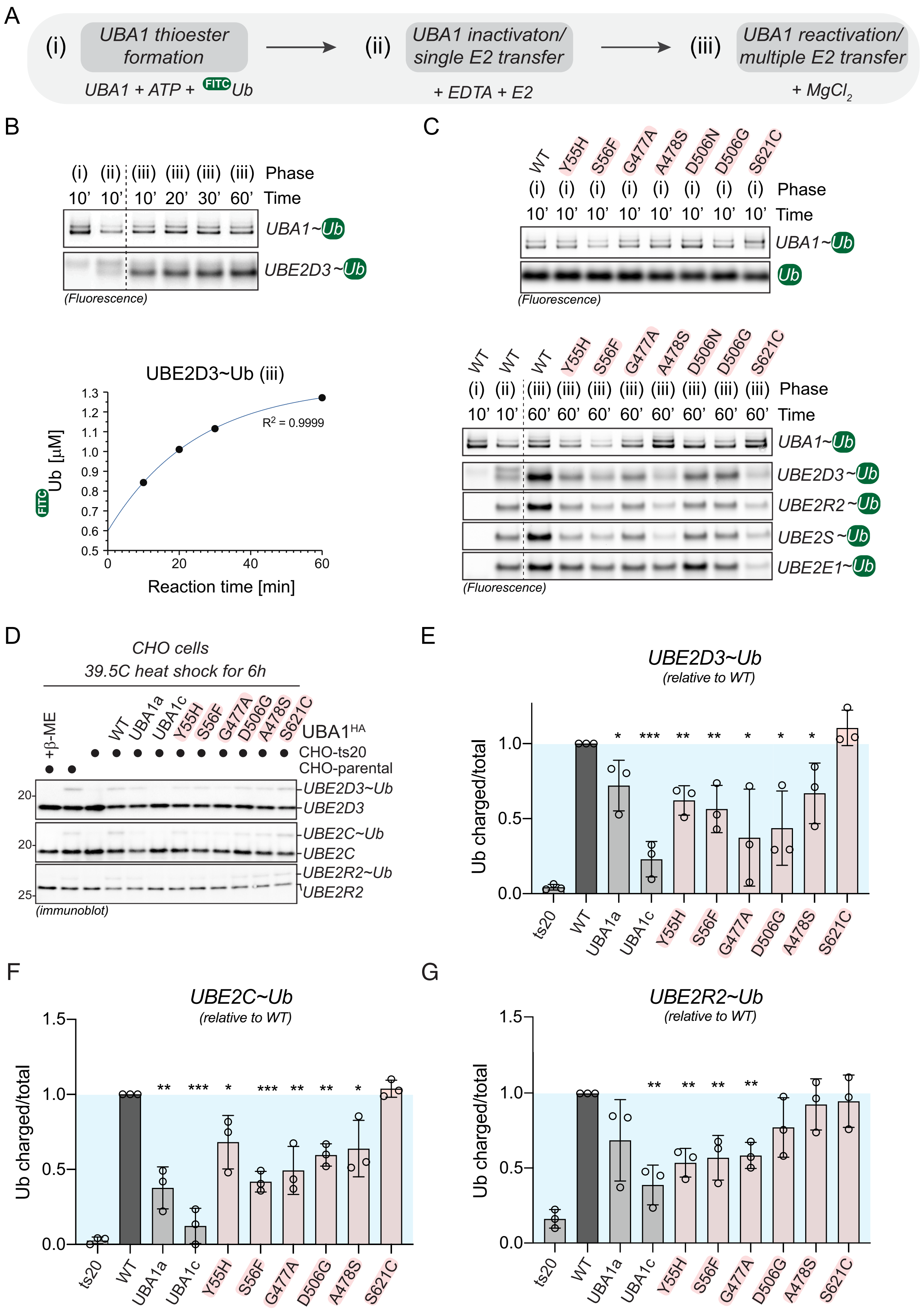
A
(i) UBA1 thioester formation
UBA1 + ATP + FITC Ub
(ii) UBA1 inactivaton/ single E2 transfer
+ EDTA + E2
(iii) UBA1 reactivation/ multiple E2 transfer
+ MgCl2
B
Phase
Time
UBA1~Ub
UBE2D3~Ub
(Fluorescence)
UBE2D3~Ub (iii)
R2 = 0.9999
FITC Ub [µM]
Reaction time [min]
C
WT Y55H S56F G477A A478S D506N D506G S621C
Phase
Time
UBA1~Ub
Ub
(Fluorescence)
UBE2D3~Ub
UBE2R2~Ub
UBE2S~Ub
UBE2E1~Ub
D
CHO cells
39.5C heat shock for 6h
+β-ME
WT UBA1a UBA1c Y55H S56F G477A D506G A478S S621C
UBA1HA
CHO-ts20
CHO-parental
UBE2D3~Ub
UBE2D3
UBE2C~Ub
UBE2C
UBE2R2~Ub
UBE2R2
(immunoblot)
E
UBE2D3~Ub
(relative to WT)
Ub charged/total
ts20 WT UBA1a UBA1c Y55H S56F G477A D506G A478S S621C
F
UBE2C~Ub
(relative to WT)
Ub charged/total
G
UBE2R2~Ub
(relative to WT)
Ub charged/total

Figure EV4. Non-canonical VEXAS mutations are deficient in transferring ubiquitin to diverse E2 enzymes in vitro and exhibit defects in cells.

(**A**) Schematic overview of the sequential, three-phase in vitro assay used to measure UBA1 transthiolation. (i) Complete charging of UBA1 by incubation of 250 nM UBA1b with 10 µM FITC-ubiquitin and 5 mM ATP (ii) Quenching of UBA1 charging and single transfer to E2 enzyme by addition of 100 mM EDTA and 1 µM E2 enzyme (iii) Reactivation of UBA1 charging and multi-transfer to E2 enzyme by addition of 100 mM $MgCl_2$ (**B**) UBA1 WT was subjected to the experiment described in panel (**A**) using UBE2D3. Reactions were subjected to SDS page and analyzed by fluorescence imaging. *Upper panel*: Fluorescence scan showing UBA1 and UBE2D3 ubiquitin thioester levels after each reaction phase. *Lower panel*: UBE2D3 ubiquitin thioester levels in reaction phase (iii) were quantified and plotted against the reaction time, revealing that UBE2D3 is maximally charged after 60 min. (**C**) Non-canonical VEXAS mutations (red) are deficient in E2 transthiolation in vitro. Indicated UBA1 proteins were subjected to the experiment described in panel (**A**) using either UBE2D3, UBE2R2, or UBE2S. Reactions were subjected to SDS page and analyzed by fluorescence imaging. *Upper panel*: Fluorescence scan showing UBA1 charging after reaction phase (i) as control. *Lower panel*: Fluorescence scan showing UBA1 re-charging and E2 transfer after reaction phase (iii). Quantifications of 3 biological replicates are shown in Fig. 5F. (**D**) Non-canonical VEXAS mutations (red) are impaired in supporting E2 ubiquitin thioester levels in cells. CHO ts20 cells were reconstituted with indicated UBA1 variants and incubated at the permissive temperature for 6 h, followed by immunoblotting using antibodies against indicated E2 enzymes. (**E–G**) Quantification of ubiquitin charging levels of indicated E2s (charged/total) shown in panel (**D**). $n = 3$ biological replicates, error bars = s.d., $^{*}p < 0.05$, $^{**}p < 0.01$, $^{***}p < 0.001$, one-way ANOVA.

This is a U.S. Government work and not under copyright protection in the US; foreign copyright protection may apply
